# Usefulness of the Distribution of Relaxation Time Method in Electroanalytical Systems: The Case of Voltammetric Ion-Selective Electrodes

**DOI:** 10.1021/acsomega.3c08656

**Published:** 2024-02-06

**Authors:** Iván Robayo-Molina, Gastón A. Crespo, María Cuartero

**Affiliations:** † Deparment of Chemistry, School of Engineering Science in Chemistry, Biotechnology and Health, KTH Royal Institute of Technology, Teknikringen 30, SE-100 44 Stockholm, Sweden; ‡ UCAM-SENS, Universidad Católica San Antonio de Murcia, UCAM Hitech, Avda. Andres Hernandez Ros 1, 30107 Murcia, Spain

## Abstract

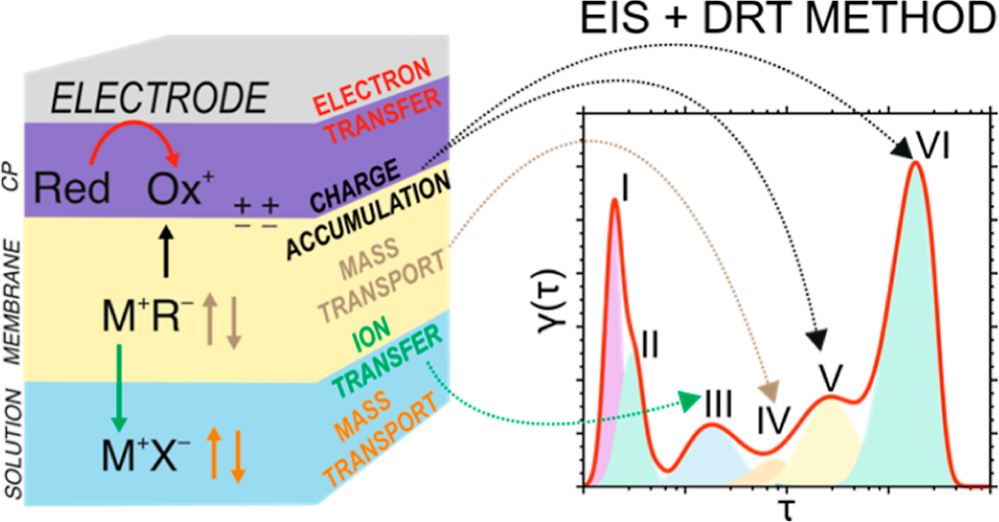

Despite the distribution of relaxation time (DRT) method providing clear insights about processes that go unnoticed by traditional electrochemical impedance spectroscopy (EIS) analysis, it has not yet been adopted to solve electroanalytical systems. As an illustration case, we apply the DRT method to deconvolve EIS data from solid-state voltammetric ion-selective electrodes (ISEs). The main aim is to shed light on the underlying working mechanism across the different materials and interfaces, specifically, the doping of a conducting polymer when covered with a very thin (ca. 230 nm) permselective membrane. Although frequency-dependent AC measurements in EIS allow the separation of processes that contribute to the electrical signal, interpretation of the data is challenging. DRT may overcome this inconvenience by revealing a series of peaks corresponding to the predominant electrochemical processes, without any preknowledge on those. To demonstrate our hypothesis, we examine the conducting polymer poly(3-octylthiophene) (POT) linked to a membrane with sodium tetrakis[3,5-bis(trifluoromethyl)phenyl]borate (Na^+^TFPB^–^) as the cation exchanger, in which the lipophilic anionic part (TFPB^–^) is responsible for the POT doping when it gets electrochemically oxidized (POT^+^). The investigation of EIS data obtained under different conditions with the DRT method showed the occurrence of several processes. We have attributed two of these to two different conformational changes in the POT film in connection with p-type charge-transfer doping. Indeed, the kinetics is found to follow a Butler–Volmer behavior, with average charge transfers of 0.5 and 0.3 mol of electrons for each peak. Overall, we demonstrate the utility of the EIS–DRT tandem to separately study charge-transfer events that interconnect along the same (interfacial segmented) system, which cannot be reached by using classical electrochemical approaches. These kinds of insights are necessary for a more efficient design and high-level exploitation of voltammetric ISEs but also other electrochemical systems such as catalysts, batteries, and photovoltaic cells.

## Introduction

Understanding all the events initiated when an interface is polarized is crucial for the rational design and proper usage of electrochemical sensors, catalysts, photovoltaic cells, and so on.^1−3^ Charge transfer, ion exchange, adsorption, coupled chemical reactions, migration, and mass transport occur at different rates and on different time scales in the interfacial domain. These processes range from the fast electron-transfer kinetics at the metal–solution interface to the slow mass transport, which typically has a diffusion coefficient of approximately 10^–6^ cm^2^ s^–1^ and takes place over time scales of seconds.^4,5^

In contrast to classical DC electrochemical techniques that depend on time (e.g., voltammetry and amperometry), nonstationary (or AC) approaches, such as electrochemical impedance spectroscopy (EIS), allow us to work at different frequencies. The key aspect is measuring the potential or current due to a transient event or periodic excitation. In essence, the transition from time-domain strategies to the frequency domain permits gaining a distinct perspective on complex electrochemical systems. For example, it is possible to deconvolve the kinetics associated with charge-transfer and mass-transfer processes or even uncouple chemical reactions.^6^ The perturbation can be applied in many forms but is usually a sinusoidal or multisine input. In the sinusoidal case, a marginal disturbance (5–10 mV) from the steady state of the system is imposed; whereas in the multisine case, the signal is the sum of multiple sine waves with different amplitudes and phases.^7^

EIS is the most broadly used nonstationary technique when studying electrochemical sensors, mainly due to its ability to provide helpful information on both reaction kinetics and transport phenomena.^7^ EIS data are typically investigated via equivalent circuit analyses^8^ or analytical models.^9^ The first path is sometimes limited because such equivalent circuits may not exist or may not yield comparable results, and with unsolved physicochemical interpretations.^10^ Moreover, the equivalent circuits’ analysis neglects ion diffusion and nonuniform ion concentrations in the electrolyte solution.^11^ On the other hand, obtaining analytical solutions for the second path may be challenging.^12^ Importantly, the distribution of relaxation time (DRT) method emerged to assist the interpretation of complex impedance spectra.^13^

DRT analysis can transform an EIS spectrum into a continuous distribution of resistances in the frequency domain. Consequently, a series of peaks corresponding to the primary relaxation times at each frequency are unveiled. Importantly, this transformation is achieved without any prior knowledge of the electrochemical processes involved in the overall system.^14^ Indeed, this is an advantage over the equivalent circuit method, which requires a detailed understanding before selecting a model that can accurately represent the electrode and device under study. DRT has been successfully used to investigate fuel cells,^15^ lithium-ion batteries,^12^ and hydrogen oxidation/reduction processes.^16^ However, to the best of our knowledge, DRT has not been yet applied to the case of systems comprising the electrochemical activation of conducting polymers (CPs, with n- or p-doping), which is relevant for supercapacitors^17^ photocatalysis,^18^ drug delivery systems,^19^ electrochemical sensors,^20^ and others. For a p-type CP, it is known that the reduced form acts as an insulator, but once the polymer is oxidized, the generation of positive bipolaron and/or polaron pairs that are delocalized over the structure chain provides conduction. Nevertheless, little is known about the kinetics of such processes, which becomes even more complicated when the CP forms part of an electrochemical device and interconnects to several charge-transfer processes occurring simultaneously or in series.^21,22^ This is the case in all-solid-state ion-selective electrodes (ISEs),^23−25^ in which the CP acts as the ion-to-electron transducer when interfacing a conductive electrode and a permselective membrane.^26,27^ The materials most used for this purpose have been poly(3,4-ethylenedioxythiophene), poly(3-octylthiphene) (POT), and polypyrrole.^28^

**Figure 1:**
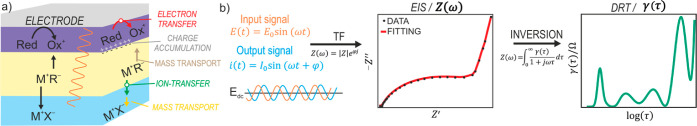
(a) Schematics of the system to be described by the DRT–EIS approach: a redox element sandwiched between a conductive electrode and a membrane containing a doping element, with the membrane immersed in an aqueous electrolyte solution. Polarization induces a series of interconnected charge-transfer processes that are to be distinguished as separate peaks in the DRT. (b) Illustration of EIS and DRT data obtained with the system described in (a). The input and output signals (voltage and current) are sinusoidal functions with a defined potential value (*E*_dc_), amplitude, and adjustable frequency during the EIS experiment. The transfer function allows one to obtain the impedance spectrum as a function of frequency. Then, by computing for γ(τ) and solving the integral (inversion), the DRT can be obtained.

In voltammetric all-solid-state ISEs, the CP is oxidized by an anodic potential sweep to generate a positive charge that is further stabilized by a lipophilic anion present in the membrane phase (Figure 1a). Accordingly, there is a series of interconnected charge-transfer processes: (i) electron transfer at the substrate–CP interface, (ii) charge transport across the CP, (iii) ion exchange at the CP–membrane interface, (iv) ion transport along the membrane, and (v) ion transfer at the membrane–water (electrolyte) interface. Altogether, these generate a current peak whose position on the potential scale depends on the associated ion transfer, being exploited for various analytical purposes.^29−31^ Notably the ion transfer purely refers to an interfacial phenomenon that depends on the applied potential and requires an activation energy.^32^ Despite some mechanistic insights being already established for the voltammetric all-solid-state ISEs,^33−35^ it is not entirely understood. One of the difficulties in unravelling the kinetics is that they occur simultaneously in four different phases (electrode, CP, membrane, and sample), being events of different natures (oxidation of the CP, doping, mass transport, chemical reactions, and electron and ion transfers) and presenting different rates.

In this paper, we investigate EIS data from voltammetric CP-membrane ISEs using DRT. Specifically, the CP is electropolymerized POT and the membrane is a very thin element (ca. 230 nm) based on sodium tetrakis[3,5-bis(trifluoromethyl)phenyl]borate (NaTFPB) as the cation exchanger. The deconvolutions of EIS spectra, observed at different membrane and electrolyte compositions, as well as POT physical characteristics, allow us to obtain unprecedented information about each of the interconnected charge-transfer processes in the system (Figure 1b). We observed a maximum of six peaks at different frequencies. These peaks are ascribed to the constant of the electrochemical cell, ion transfer at the membrane–sample interface, charge transfer across the membrane domain, and several conformational configurations of the POT regarding the p-doping event, among others. The DRT–EIS approach developed here is expected to shed light on not only the mechanism underlying voltammetric ISES but also other electroanalytical and electrochemical systems that rely on activating CP or other semiconducting materials.

## Experimental Section

### Reagents

All aqueous solutions were prepared in ultrapure water (Millipore Milli-Q, specific resistivity 18.2 MΩ). Lithium perchlorate (>98%, LiClO_4_), 3-octylthiophehe (97%), high-molecular-weight poly(vinyl chloride) (PVC), bis(2-ethylhexyl)sebacate (DOS), NaTFPB, potassium chloride (99.5%, KCl), tetrahydrofuran (>99.9%, THF), and acetonitrile (>99.8%, ACN) were purchased from Sigma-Aldrich.

### Preparation of POT–Membrane Electrodes

A solution of 0.1 M 3-octylthiophene/0.1 M LiClO_4_ in ACN was used for POT electropolymerization on glassy carbon electrodes (GCEs, area = 0.1964 cm^–2^, Metrohm RRDE.GCPT.S). Galvanostatic conditions were employed (e.g., constant current density of 0.89 mA cm^–2^ for 20 s), and, subsequently, the film was discharged at 0 V for 240 s in 0.1 M LiClO_4_/ACN solution. A platinum electrode (Metrohm, 6.0331.000) was used as the counter electrode, and homemade Ag/AgCl wire was used as the reference electrode (RE). These electrodes were also utilized for any electrochemical measurement together with a Vionic potentiostat (Metrohm Autolab B.V.) controlled by Intello 1.2 software. Notably, the RE was selected to count on a low resistance in the electrochemical cell. On top of the POT film, a permselective membrane was formed by spin coating (30 μL, 1400 rpm) a cocktail solution containing the polymer PVC (1.87 mg), plasticizer DOS (3.75 mg), and a variable concentration of NaTFPB (0.22, 0.44, 0.66, or 0.88 mg, corresponding to 40, 80, 120, and 160 mmol kg^–1^ of the membrane) dissolved in 0.5 mL of THF. The very same membrane was fully characterized by our group (e.g., atomic force microscopy, scanning electron microscopy images, X-ray photoelectron spectroscopy, and ellipsometry measurements) in previous studies.^36,37^

### Electrochemical Impedance Spectroscopy

EIS measurements were performed by using a three-electrode system in 10 mM KCl solution. Data were acquired in the frequency range from 250 kHz to 50 mHz, with the AC amplitude of the sinusoidal excitation signal being 10 mV. Before running the EIS measurements, we conducted a cyclic voltammetry experiment (several scans) to obtain a precise value of *E*_peak_. Then, EIS was always carried out at that *E*_peak_ ± 100 mV in steps of 20 mV. Any possible variation in the reference potential because of the changing chloride content was corrected by referring all measurements to the corresponding *E*_peak_ in the voltammogram. The spontaneous exchange of K^+^ by Na^+^ in the electrode upon the first contact with the sample solution is expected. Notably, this process is extremely fast (ms) in very thin membranes, as calculated elsewhere.^31^

### Distribution of Relation Times

As illustrated in Figure 1b, the DRT method for analyzing EIS data requires the solution of the Fredholm integral equation.^38^ The relation between the impedance (*Z*) and the DRT function (γ) is described by eq 1
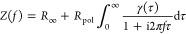
1where *R*_∞_ is the Ohmic resistance, *R*_pol_ the polarization resistance, τ is the relaxation time, and *f* is the frequency. Several approaches to obtaining the solution of such an integral have been reported, including spectral division,^39^ regularization techniques for deconvolution,^40^ and direct solution of the Fredholm integral.^41^ Solving eq 1 is an “ill-posed inverse problem” because the solution does not depend continuously on its parameters. In this paper, we apply *DRTtools*, a free toolbox developed by Ciucci et al.,^40^ for computing DRTs from EIS data via the Tikhonov regularization (more details are provided in the Supporting Information, Figures S1 and S2 and Tables S1 and S2). Notably, a regularization parameter (λ) equal to 10^–3^, which is a common value found in the literature for Gaussian decomposition,^40^ was selected. Further data processing concerning peak separation, detection of peak maximum frequency, and area calculation were carried out using a homemade code in *R* Project for Statistical Computing, version R4.1.2.^42^

## Results and Discussion

### Voltammetric Behavior of the System

**Figure 2:**
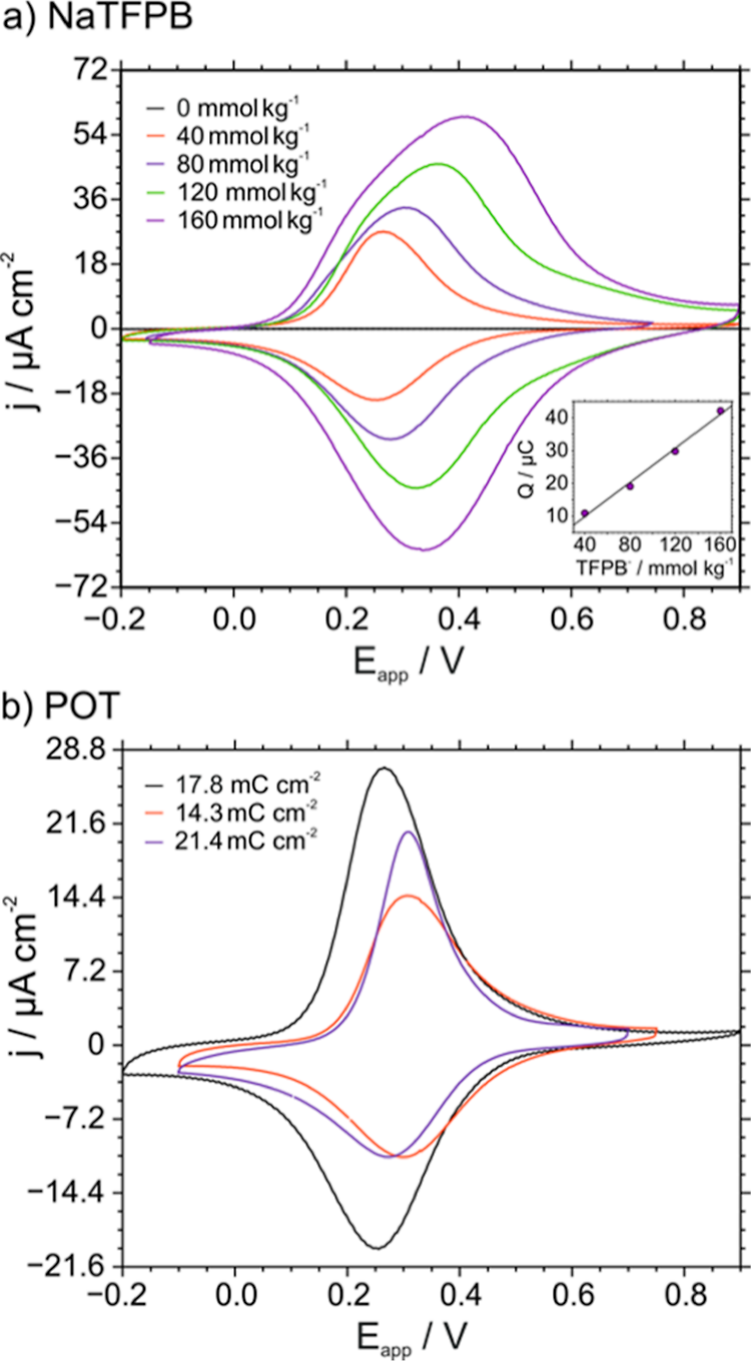
Cyclic voltammograms obtained in 10 mM KCl for the POT|membrane system under different conditions: (a) increasing concentrations of NaTFPB in the membrane for a POT film synthesized with 17.8 mC cm^–2^ and (b) increasing the charge density during the electrodeposition of POT. Scan rate = 100 mV s^–1^. NaTFPB = 40 mmol kg^–1^.

An ISE consisting of a redox-active film (such as POT) connected to a thin permselective membrane can be described by an electron-transfer–ion-transfer scheme (ET–IT).^36,43^ When a potential sweep is applied in the positive direction, a series of ITs at the membrane–water interface are generated driven by electroneutrality maintenance. The ITs are manifested as voltammetric peaks, whose potential (*E*_peak_), current, and charge may vary with both the membrane composition and POT layer configuration in the electrode. Accordingly, we investigated the voltammetric response of several ISEs prepared with membranes that comprise increasing amounts of NaTFPB (0, 40, 50, 120, and 160 mmol kg^–1^ of the membrane) and increasing charge of the electrodeposited POT layer (14.3, 17.8, and 21.4 mC cm^–2^ corresponding to applied current densities of 0.71, 0.89, and 1.07 mA cm^–2^ in the galvanostatic electrodeposition for 20 s). Figure 2 depicts the results.

As a general trend, rather reversible waves appeared (Table S3): the anodic peak corresponding to the oxidation of POT (ET) coupled with a cation release (IT) from the membrane to the solution and then the corresponding cathodic peak ascribed to the reduction of POT (ET) and a cation uptake (IT) from the solution to the membrane. Importantly, the charge involved in the interconnected events must be the same, i.e., the charge generated in the POT lattice as per its oxidation is equal to the charge transferred at the membrane–water interface and vice versa.

The charge was found to increase with increasing TFPB^–^ concentrations in the membrane, keeping the POT film to a constant electrodeposited charge of 17.8 mC cm^–2^ (Figure 2a and Table S3). Also, the peaks became wider and tended to lose their Gaussian-like shape. Overall, the results indicated that the concentration of the cation exchanger in the membrane sets the charge involved in the ET–IT process. Indeed, the relationship between the NaTFPB concentration in the membrane and the charge under the voltammetry peaks was found to be linear (inset in Figure 2a). Then, it seems that the insertion mechanism of TFPB^–^ into the POT lattice (i.e., p-doping) ultimately affects the shape of the CVs. Noteworthy, at the selected experimental conditions and assuming some membrane parameters, the amount of TFPB^–^ involved in such a doping constitutes ca. 65% of the available TFPB^–^ in the membrane (see Table S4).

Varying the current density applied during the galvanostatic electrodeposition of POT was found to modify the voltammetric peak (Figure 2b). We measured the potential during the electropolymerization. The final voltages were 1.462, 1.414, and 1.406 V for current densities of 0.71, 0.89, and 1.07 mA cm^–2^, respectively. The variation in the final potential among the different current densities was relatively small (∼50 mV). As a result, we did not consider an overoxidation of the POT film. However, increasing the deposited charge from 14.3 to 17.8 mC cm^–2^ generated a significant increase of the charge under the voltammetric peak (from 7.2 to 11.1 μC in the anodic wave and from 6.31 to 11.2 μC in the cathodic one). The contrary occurred when increasing the electrodeposited POT charge even more, up to 21.4 mC cm^–2^: the charge in the anodic and cathodic waves decreased to 6.78 and 6.47 μC, respectively.

A rather similar effect was previously reported,^37^ but instead of galvanostatic deposition, the authors used cyclic voltammetry technique from −0.5 to 1.2 V. They observed that ∼2 full cycles (−0.5 to 1.2 and 1.2 V to −0.5 counts as one cycle) were the optimal conditions in terms of the magnitude for the ion-transfer peak current. Above two cycles (thicker films but less compacts), the peak current diminished considerably. It is known that an increase in the applied current density for an electrodeposition process generally increases the thickness of the layer but also results in less compact films, changes in the morphology, and even inhomogeneities, any of which may affect the electrochemical signal of the material under study.^44,45^ In the case of POT, the current reduction may be attributed to an increase in the surface roughness, which diminishes the electrochemical reactivity and/or inhibits the p-doping.^46^

### Electrochemical Impedance Spectroscopy

**Figure 3:**
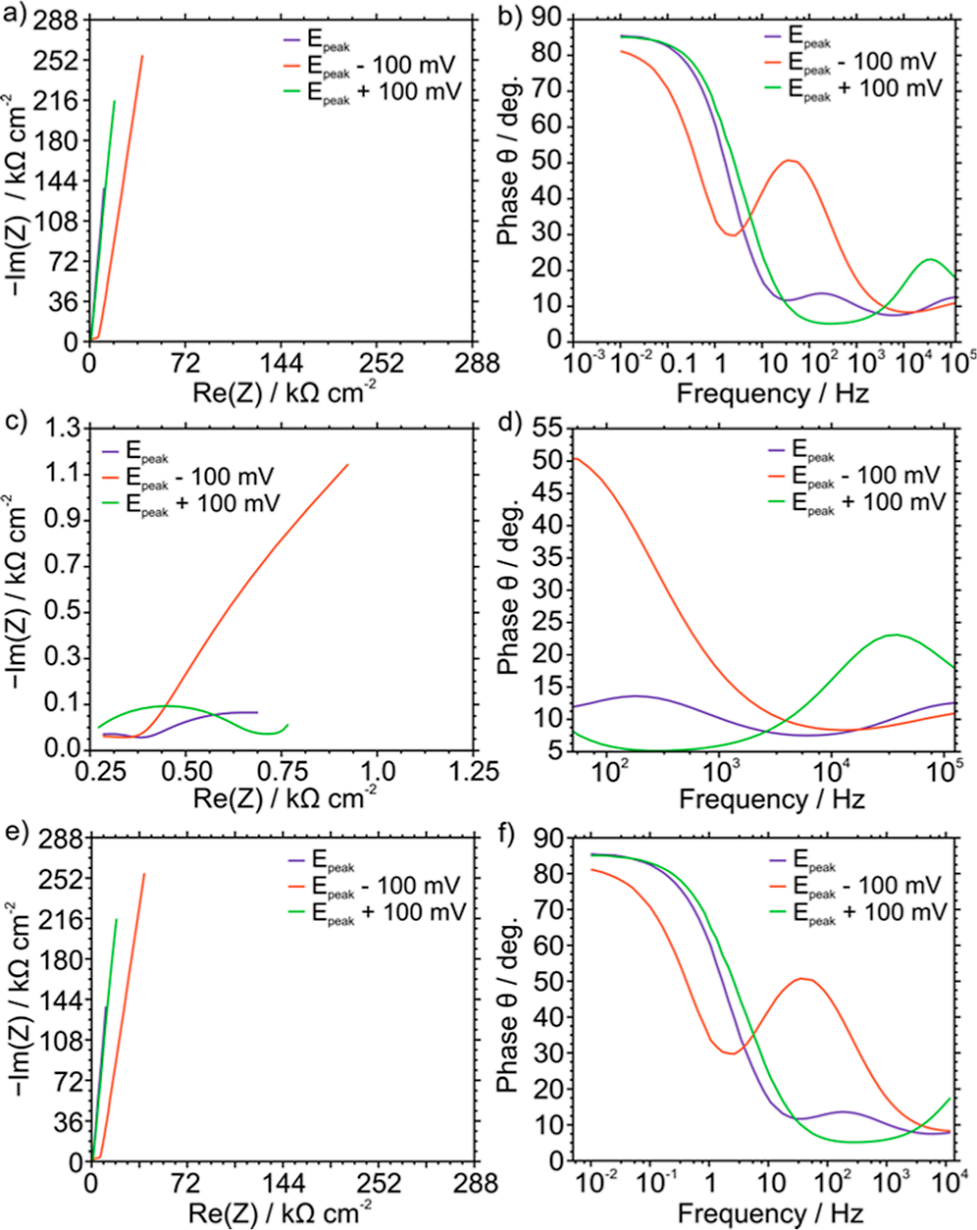
Impedance spectra obtained at different *E*_dc_ inputs (*E*_peak_ ± = 100 mV). The reference *E*_dc_ was selected equal as the *E*_peak_ obtained from a CV experiment conducted before the EIS experiment. The electrode consisted of a POT film (17.8 mC cm^–2^) and a membrane containing 40 mmol kg^–1^ NaTFPB. Background electrolyte: 10 mM KCl. Left: Nyquist plot. Right: Bode plots. Frequencies: (a) and (b) full spectrum, (c) and (d) from 100 kHz to 50 Hz, and (e) and (f) from 14 kHz to 50 mHz.

Considering the case of a membrane comprising a concentration of 40 mmol kg^–1^ NaTFPB and a POT layer with an electrodeposited charge density of 17.8 mC cm^–2^, we obtained EIS spectra in an *E*_dc_ window of ±100 mV with respect to the *E*_peak_ displayed in the CV in 10 mM KCl solution (i.e., 265 mV). The results, expressed as Nyquist and Bode plots, are presented in Figure 3 for *E*_dc_ equal to *E*_peak_, *E*_peak_ – 100 mV, and *E*_peak_ + 100 mV. 3D plots considering *E*_dc_ at each 20 mV (except for the two lowest potentials) are presented in Figure S3. The real and imaginary components of the impedance were observed to be minimized when *E*_dc_ coincided with the voltammetric *E*_peak_. Reproducibility was assessed by means of data provided by three analogous electrodes (Figure S4). A Kramers–Kronig (K–K) test (Figure S5) revealed that the discrepancy between the measured impedances and the K–K transform is less than 5%, confirming the reliability of the data. In addition, one can be assured that the response of the system is due to the potential stimuli and not to the system spontaneously evolving into a different state.

In the diffusion region, the Bode plots exhibited a phase angle exceeding 80°, with a resistance greater than 500 KΩ (2.55 MΩ cm^–2^) in the Nyquist plot at all points. This suggested a capacitor-like behavior, a phenomenon previously reported for CP^47^ and that is associated with the absence of a flux at the corresponding boundary, i.e., (∂*C*/∂*x*)_*x*=0_ = 0. Then, the deviation in the phase angle from the theoretically ideal capacitor (90°) is often attributed to surface inhomogeneities.^48,49^ Consequently, mass transport is blocked at the outer edge of the film or the polymer/electrolyte interface.^47,50^ Accordingly, the overall charge transfer in the system will not be limited by diffusion in any of the phases, which is a consequence of the thickness of both the POT and the membrane layers and also of the high electrolyte concentration.

The two semicircles in the Nyquist and Bode plots at the *E*_dc_ equating to the *E*_peak_ are associated with two different charge-transfer processes in the system. These semicircles were found to shift toward higher frequencies and to diminish when approaching *E*_peak_, indicating faster kinetics. For example (see Figure S3), the first semicircle shifted from 72.7 Hz (at 0.05 V) to 1.20 kHz (at 0.32 V), and the second semicircle shifted from 75.3 kHz (at 0.5 V) to 385 kHz (at 0.32 V). These semicircles are significant on both sides of *E*_peak_: the first semicircle at lower potentials and the second at potentials above *E*_peak_.

**Figure 4:**
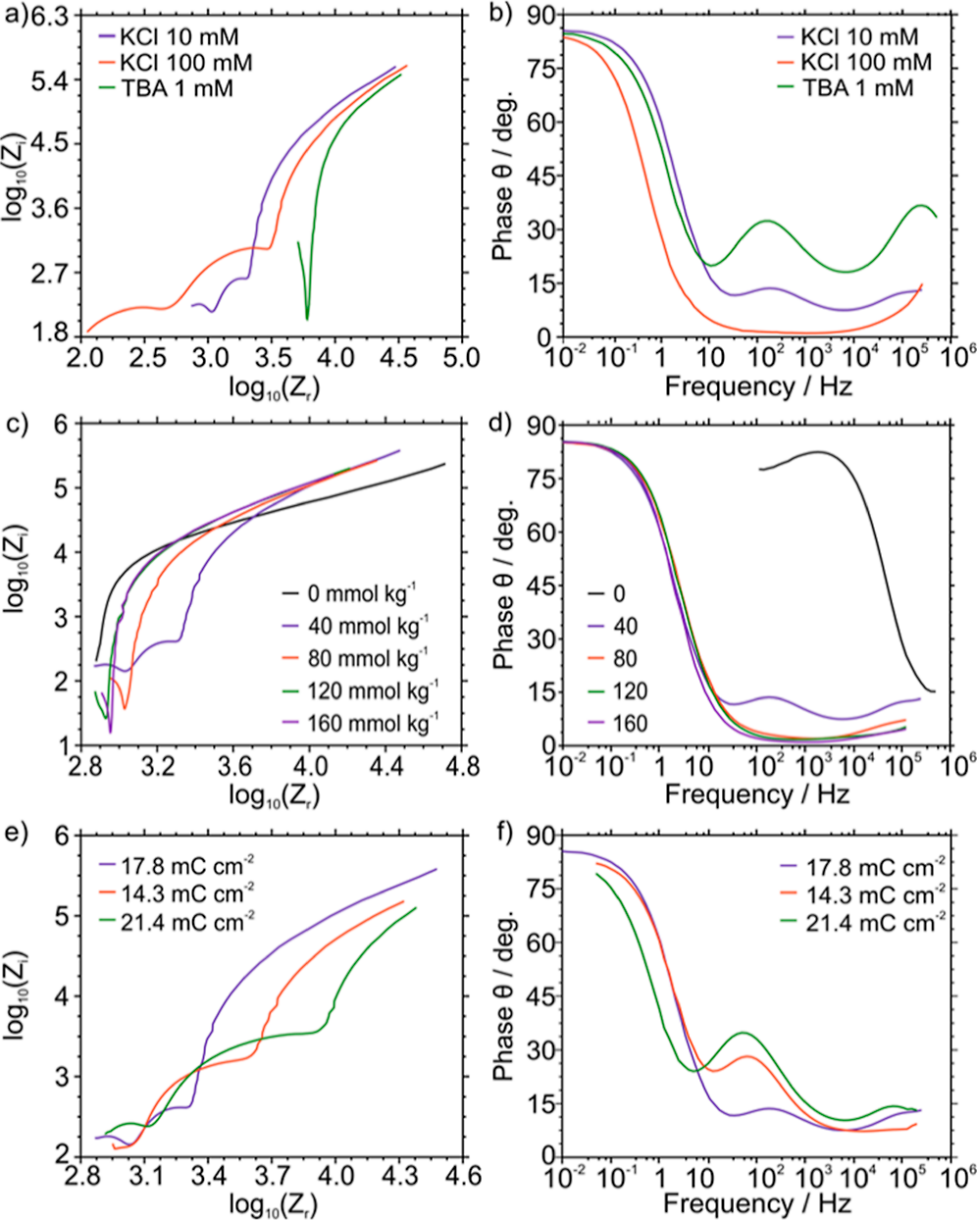
Impedance spectra obtained under different conditions, considering an excitation potential (*E*_dc_). Left: Nyquist plots. Right: Bode plots. (a) and (b) Show the change in the electrolyte; (c) and (d) show the change in the TFPB^–^ concentration in the membrane; and (e) and (f) show the variation of the POT.

Figure 4 shows the logarithms of the real and imaginary impedance over the frequency range for a better comparison of the results under different conditions (i.e., changing the electrolyte, the NaTFPB in the membrane, and the electrodeposited POT charge). A particularity of this sort of representation is the distortion of the semicircles and the diffusion region, which must be considered to draw appropriate conclusions. Increasing the concentration of the electrolyte solution, regardless of the cation nature, translated to a decrease in resistance (Figure 4a,b). Then, based on the Bode plot (Figure 4b), the electrolyte concentration did not remarkably affect the position (frequency) of the semicircles.

The TFPB^–^ concentration in the membrane was found to significantly influence the EIS (Figure 4c,d). No charge processes are present without any TFPB^–^, a conclusion based on the absence of semicircles. Then, the phase angle at low frequencies tended to be almost 90°, a behavior that resembles that of a capacitor. Another feature to highlight is the considerably high resistance compared to membranes containing TFPB^–^. For example, at a frequency of 50 mHz, the resistance was 225 MΩ cm^–2^, while in the presence of TFPB^–^, the resistance is less than 54 kΩ cm^–2^ at all the concentrations (41.8, 28.5, 19.8, and 17.2 kΩ cm^–2^ for 40, 80, 120, and 160 mmol kg^–1^, respectively). Furthermore, the two semicircles in the charge-transfer region became smaller as the TFPB^–^ concentration increased, with the Bode plots showing a decrease in the height of the angle phase. This indicated faster kinetics with increasing TFPB^–^. Then, regardless of the TFPB^–^ concentration, the phase angle tends to reach 90° at lower frequencies. Therefore, in the range of 0.5 Hz–50 mHz, the impedance can be described by a simplified RC circuit, comprising a resistor in series with a capacitor,^51^ and the imaginary part of the complex impedance (*Z*_im_) can be expressed as
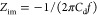
2where *C*_d_ is the equilibrium differential capacitance.

A deeper evaluation of the data (3D plots of the ESI data obtained under the different electrolyte, TFPB^–^, and POT conditions, Figure S6) confirmed that −*Z*_im_ is inversely proportional to the frequency (Figure S7). Then, the values of *C*_d_ determined at the voltammetric *E*_peak_ using eq 2 are collected in Table 1. The capacitance was found to linearly increase with the TFPB^–^ concentration (see Figure S8), and hence, it can be concluded that this is the primary ion participating in the capacitor-like part of the system, also revealing that this occurs at the polymer–membrane interface.

**Table 1:** Values for the Differential Capacitances Calculated at Increasing TFPB^–^ Concentrations in the Membrane^a^

TFPB^–^/mmol kg^–1^	0	40	80	120	160
*E*_peak_/mV	400	260	290	360	380
*C*_d_/μF cm^–2^	1.8 × 10^–3^	13.9	20.0	25.8	32.6

a Residual standard deviations for the measurements were found to be <10% (*n* = 3 Electrodes).

Regarding the effect of varying the electrodeposited POT charge, the presence of two semicircles was evident in all the cases. However, we did not find a clear relationship between the resistance and the charge density from the electrodeposition of POT. Going from 14.3 to 17.8 mC cm^–2^ translated to a decrease in the resistance but further increasing the charge density up to 21.4 mC cm^–2^ resulted in a significant increase in the resistance. As mentioned, there is an optimal current to be applied in the galvanostatic POT electrodeposition to generate a suitable film in terms of charge, thickness, and morphology that facilitate both the ET and p-doping events.

### Implementation of the DRT Method

**Figure 5:**
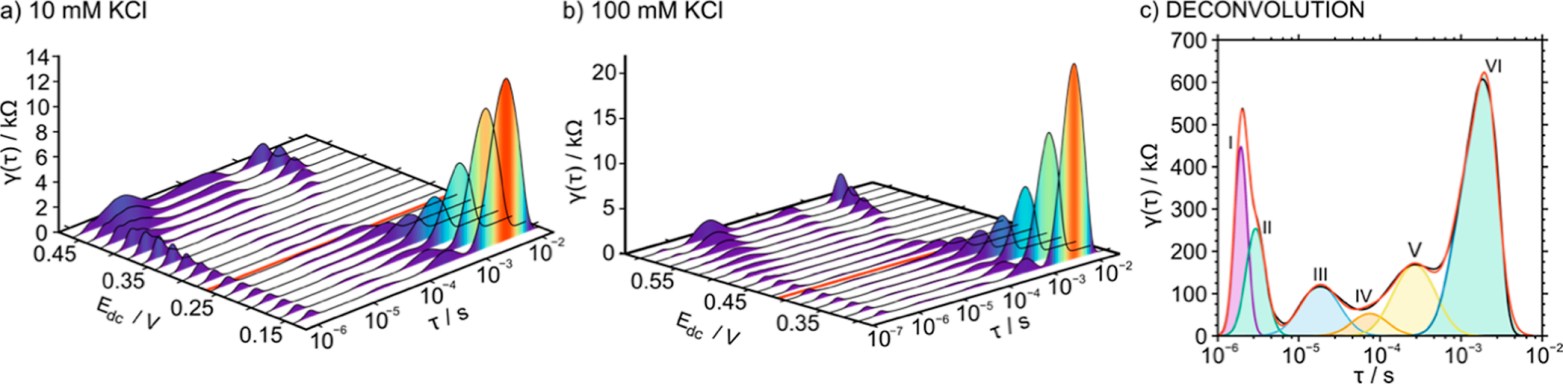
(a,b) DRT functions calculated from impedance spectra obtained at varying *E*_dc_ with respect to the voltammetric *E*_peak_ (red line). The electrode consisted of a POT film (17.8 mC cm^–2^) and a membrane composed of 40 mmol kg^–1^ NaTFPB immerse in (a) 10 mM KCl, *E*_peak_ = 260 mV and (b) 100 mM KCl, *E*_peak_ = 410 mV. (c) Example of DRT deconvolution for the case of the POT film (17.8 mC cm^–2^), membrane composed of 40 mmol kg^–1^, 100 mM KCl electrolyte, and *E*_dc_ corresponding to the *E*_peak_ (410 mV).

Figure 5a,b depicts 3D plots of the DRT functions γ(τ) calculated from the EIS data obtained in 10 and 100 mM KCl solutions, respectively: the DRT function is plotted against *E*_dc_ and τ on a logarithmic scale from 100 kHz to 1 Hz. Lower frequencies in the region of capacitor-like behavior were not considered to facilitate observation of the DRT peaks at medium–high frequencies. Overall, the finite space diffusion utilized in the DRT method results in the likelihood of revealing a series of peaks in connection with the different physical and electrochemical processes occurring in the system. This is expected to provide insights into the kinetics and resistance of the linked events in turn.

Each process rate is described by the maximum frequency of the corresponding peak, and the DRT resistance can be determined by the area over the logarithmic τ scale. In our case, τ and the resistance at each DRT peak were found to change with *E*_dc_. As a general trend, the peaks tended to shift toward higher frequencies and to decrease their area when *E*_dc_ approaches the voltammetric *E*_peak_. In many cases, the peaks were found to overlap, and a Gaussian deconvolution was additionally applied to the study, as exemplified in Figure 5c. Thus, a maximum of six peaks were identified considering all the tested experimental conditions, labeled from now on as I–VI in the order of increasing frequency at which they appeared. Notably, peak II normally appears as a shoulder of peak I. The τ and DRT resistance values observed for peaks I–VI are collected in Tables S5–S10.

Considering the evolution of these peaks under the different experimental conditions, we can divide them into two groups. In the first group (peaks III–VI), τ decreases when *E*_dc_ approaches to the voltammetric *E*_peak_, whereas in the second group (peaks I and II), τ is barely affected by *E*_dc_ (Tables S5–S7). Accordingly, peaks I and II (shoulder of peak I) seem related to the same process, which is likely related to the *RC* constant of the cell (*R* being the Ohmic resistant of the system and *C* the geometrical capacitance), since this is not affected by *E*_dc_ in the impedance experiment. On the other hand, it is expected that the events linked to peaks III–VI involve different charge-transfer processes, becoming faster as *E*_dc_ approaches the voltammetric *E*_peak_.

**Figure 6:**
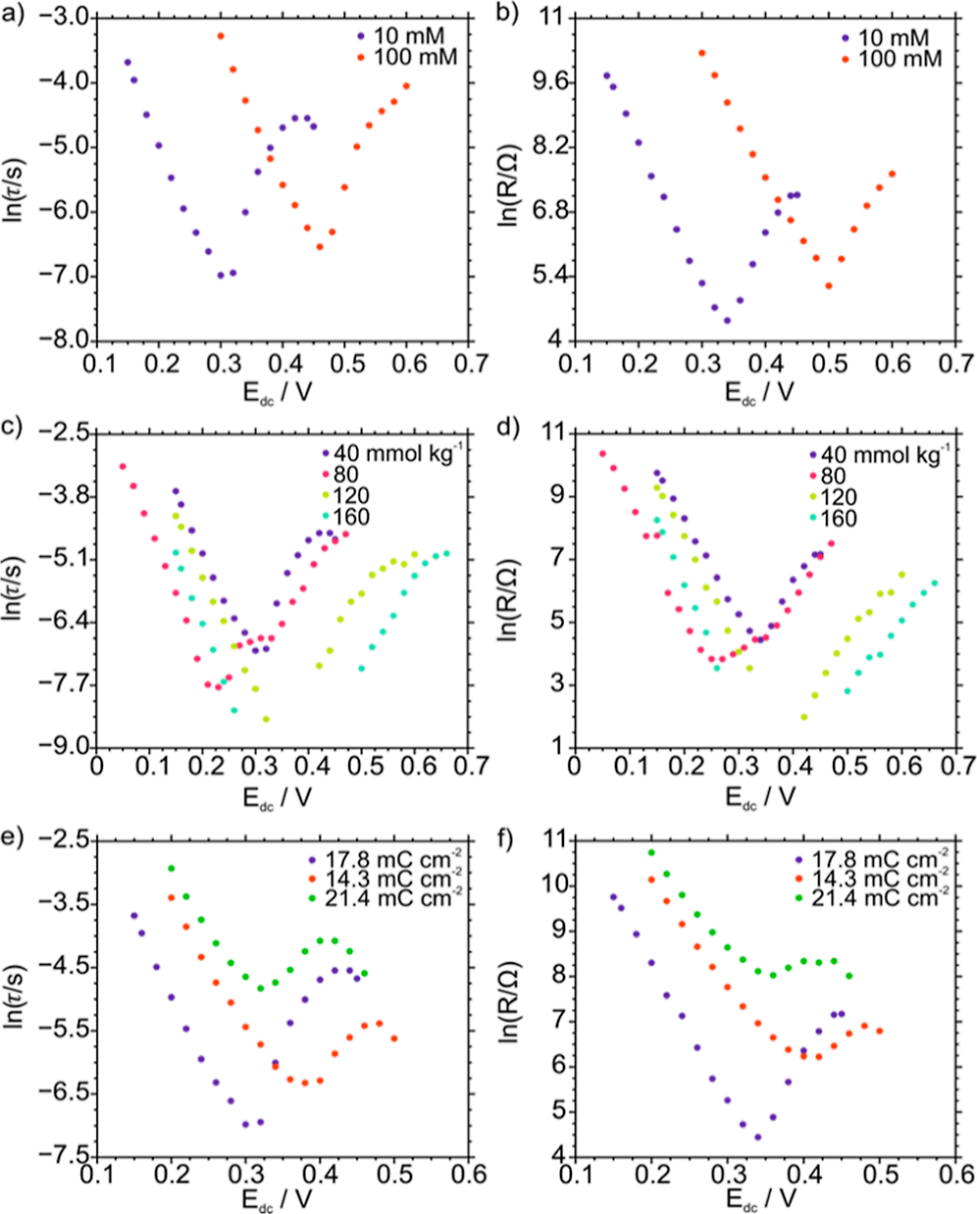
For peak VI, plot of the logarithm of the relaxation time (left column) and DRT resistance (right column) as a function of *E*_dc_ and (a,b) the electrolyte concentration in the sample, (c,d) the TFPB^–^ concentration in the membrane, and (e,f) the charge density in the POT electropolymerization.

The resistance was found to decrease as *E*_dc_ approaches the voltammetric *E*_peak_ (Tables S8–S10), i.e., the charge-transfer processes experience a significant decrease in resistance under more favorable conditions. For a deeper inspection, the logarithms of τ and DRT resistance versus *E*_dc_ were plotted. This type of plot unravels the sorts of kinetics involved in these processes. The results for peak VI are presented in Figure 6, while those for the other peaks are provided in Figures S9–S13.

The increase from 10 to 100 mM in the KCl concentration of the electrolyte (Figure 6a,b) was found to have an effect on the position of the V-shaped curves obtained for both τ and the DRT resistance. In essence, both properties decreased when increasing *E*_dc_ up to the voltammetric *E*_peak_, and they started increasing when passing beyond it. Moreover, the linear relationship between the logarithm of τ and the *E*_dc_ indicates Butler–Volmer kinetics,^52^ linked to the occurrence of Faradic process(es). The values of τ and the DRT resistance were slightly lower for 10 mM KCl (0.930 ms and 192 Ω) than for 100 mM KCl (1.45 ms and 260 Ω), but these differences were not significant enough to conclude that the process(es) behind peak VI are influenced by the electrolyte concentration in the sample solution. The minimum values for the two properties reached at a lower potential *E*_dc_ = *E*_dc,min_ for 10 mM KCl than for 100 mM KCl. This coincides with the fact that the voltammetric peak appears at an increasing *E*_peak_ for increasing KCl concentrations in the solution.^53^ Acknowledging that we are looking into a series of interconnected charge-transfer processes ultimately depending on cation release from the membrane to the solution, this latter process will be more difficult (in energy terms) at a higher concentration of the cation in the solution, translating into a higher voltammetric *E*_peak_. Notably, *E*_dc,min_ was always slightly higher than *E*_peak_.

Regarding the amount of NaTFPB present in the membrane (Figure 6c,d), similar curves and trends were observed as with the aqueous electrolyte concentrations. The increase in *E*_dc,min_ with the NaTFPB concentration relates to the shift in the voltammetric *E*_peak_ to higher values (Figure 2a). However, both τ and DRT resistance presented at *E*_dc,min_ were found to change, decreasing with the NaTFPB in the membrane, especially at 120 and 160 mmol kg^–1^. Then, changing the configuration of the POT layer (Figure 6e,f) resulted in a significant increase in both τ and DRT resistance, revealing a clear dependence together with the Faradaic nature. Effectively, at *E*_dc,min_, values of 1.79, 0.93, and 7.98 ms were obtained for τ, while 591, 192, and 4337 Ω were calculated for the resistance: we see here a decrease of the parameters from 14.3 to 17.8 mC cm^–2^, with further increase when increasing the charge density up to 21.4 mC cm^–2^. Peak VI relates to the POT doping with TFPB^–^, being of a Faradaic nature (linearity with increasing *E*_dc_, Butler–Volmer kinetics). While increasing TFPB^–^ always favors the process, there is an optimal POT configuration provided by the charge density equal to 17.8 mC cm^–2^.

The behavior of peak VI was also shown by peak V (Figure S13), so it can be concluded that both peaks are linked to POT doping by TFPB^–^. However, peak V refers to a relatively faster and less resistive process. Accordingly, it is observed that the DRT is able to discern between two different POT conformations affecting the overall kinetics of the p-doping process. Some calculations can be accomplished in this regard, as explained below. It is known that the electrochemistry of CPs (including POT) involves reversible swelling/shrinking events.^54^ The oxidation process implies the repulsion of neighbor chains close to where the positive charge is created. This repulsion induces a conformational change in the polymer to generate free volumes (or spaces) between the chains so that a counteranion can access the polymer lattice to balance the positive charges on the chains.

In 0.1 M LiClO_4_/ACN solution, this process can be described by the empirical oxidation rate, as given in eq 3(46)
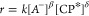
3where *r* is the oxidation rate, *k* is the rate constant, β and δ are the reaction orders, [CP*] is the concentration of active centers in the CP, and [*A*^–^] is the concentration of the counteranion. When the process follows Butler–Volmer kinetics,^54^ as is suggested by our results, *k* in eq 4 is defined as
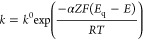
4where *k*^0^ is the standard rate constant, *E* is the electrode potential, *E*_q_ is the equilibrium potential, α is the symmetric factor, *Z* is the number of electrons transferred in the electrochemical reaction, *F* is the Faraday constant, *R* is the gas constant, *T* is the temperature, and α is the charge-transfer coefficient.

The electrochemically stimulated conformation relaxation (ESCR) model assumes that the rate at which conformational changes occur in a CP film immersed in an electrolyte media follows a Butler–Volmer-type kinetics.^52^ Accordingly, τ can be described by eq 5, considering a constant temperature
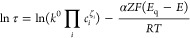
5where *C*_*i*_^ζ^_*i*_ refers to the concentration of each interfacial species (i) and ζ_*i*_ is the stoichiometric (or empirical) coefficient.

Equation 5 is following extrapolated to the case that the CP (i.e., POT) is in contact with a membrane based on an electrolyte that can be doped with the oxidized CP^+^, like the situation in the solution phase. By plotting ln τ vs *E*_q_ – *E* (*E*_q_ corresponds to the potential at which τ is minimum) only in the first part of the V-shaped curves (Figure 6), we obtained information about the charge transfer from the slope of the linear fitting, whereas the intercept relates to the apparent standard rate (*k*^0′^ = *k*^0^ ∏_*i*_*C*_*i*_^ζ^_*i*_). Consequently, DRT results were further analyzed using eq 5 to obtain deeper insights into the system. Table 2 collects the slope, the intercept, and the coefficient of determination (*R*^2^) resulting from the linear regression between ln τ and *E*_q_ −*E* derived from eq 5.

**Table 2:** Values of the Parameters in eq 5 Obtained from the Linear Regression of ln(τ) Versus *E*_dc_ at Increasing NaTFPB in the Membrane

peak	NaTFPB (mmol kg^–1^)	slope (C J^–1^)	intercept	*R*^2^	*k*^0′^ (cm s^–1^)	*Z*
VI	40	–9.64	–0.206	0.992	0.814	0.48
VI	80	–11.93	–0.226	0.992	0.797	0.59
VI	120	–10.60	–0.234	0.999	0.791	0.53
VI	160	–12.73	–0.250	0.999	0.778	0.63
V	40	–6.43	–4.71	0.916	0.009	0.32
V	80	–10.17	–1.43	0.972	0.239	0.51
V	120	–6.09	–1.94	0.927	0.144	0.30
V	160	–6.42	–2.03	0.922	0.131	0.32

Linearity was additionally verified with quantile–quantile (Q–Q) plots (Figures S14 and S15 in the Supporting Information). Values of *Z* (assuming a typical value of *a* = 0.5) and *k*^0′^ were calculated from the slopes and intercepts. On average, the charge transfer was less than a mole of electrons, ca. 0.5 for peak VI and 0.3 for peak V. Assuming that peaks VI and V are the only ones connected to the POT oxidation process (which is demonstrated below), the total charge transfer is about 0.8 mol of electrons, which agrees with previous reports.^33,36,43^ Regarding *k*^0′^, it is about 6 times larger for peak VI than peak V and hence the kinetics of the process behind peak VI is faster than that in peak V.

The results for peaks V and VI were confirmed by additional chronoamperometry experiments performed at concentrations of 40 and 120 mmol kg^–1^ TFPB^–^ in the membrane (Figure S16). In the positive direction, the applied potential was set to −0.1 V for 30 s and then stepped to the desired potential, which was increasingly approaching the voltammetric *E*_peak_. The current profiles presented a shoulder at a time before 100 ms. This behavior is attributed to an initial nucleation/relaxation in the POT film when the oxidation starts from a conformational packed film, as described by the ESCR model.^46^ Then, the current decay seems to be enhanced by increasing the TFPB^–^ concentration, i.e., faster kinetics. In the cathodic direction, the current exhibited a Cottrellian behavior without any visible shoulder. Overall, any difference detected between the results from the positive and negative directions can be associated with the higher reorganization energy involved in the transition from the compact POT structure in its reduced state to a more open structure in the oxidized state rather than vice versa.^55^

Unlike peaks V and VI, the values of τ and DRT resistance for peaks III and IV did not present as clear V-shape trend with increasing potential (Figures S11 and S12), and the values are relatively lower, viz. some tens of microseconds for τ and a few hundred Ohms for DRT resistance. For example, using 100 mM KCl, τ values were calculated to be 8.61 and 27.5 μs, and the resistances were 140 and 100 Ω for peaks III and IV at the corresponding *E*_dc,min_ (red points in Figures S11a,b and S12a,b). Importantly, the relaxation time for peak IV is close to that previously found for the potassium transfer across the length of a permselective membrane based on dipalmitoleoyllecithin (32.5 μs).^56^

As a general trend, the event related to peak IV tends to be faster and less resistive as it nears the voltammetric *E*_peak_. For τ, the tendencies regarding KCl concentration, NaTFPB concentration, and the POT charge were unclear and not compelling. For the DRT resistance, the KCl concentration did not show a meaningful effect, with only a displacement of *E*_dc,min_ due to a concentration change. In contrast, the TFPB^–^ concentration was found to have a consequential effect: when it was increased, the resistance tended to decrease. For example, the calculated values at *E*_dc,min_ were 65.2, 27.9, 11.2, and 6 Ω for TFPB^–^ concentrations of 40, 80, 120, and 160 mmol kg^–1^, respectively. The charge of the POT film manifested a certain effect only when it was below the optimal value. Overall, peak IV is mainly related to the TFPB^–^ concentration in the membrane, and thus, this peak is ascribed to the charge transfer across the membrane length to generate both the POT doping and to the IT at the membrane–sample interface.

Importantly, peak III did not appear in all of the experimental conditions: (i) it was displayed at any value of *E*_dc_ only at the highest KCl concentration, (ii) it disappeared from a concentration of 80 mmol kg^–1^ NaTFPB in the membrane, and (iii) it appeared at certain *E*_dc_ values for the optimal POT charge (17.8 mC cm^–2^), presenting higher τ and DRT resistance than the other two charges that were assayed. Moreover, for these two POT charges, τ increased, and DRT resistance was relatively constant for increasing *E*_dc_. Due to this mixed dependency with the different variables, it was not possible to assign peak III to only one process occurring in the system. Indeed, this peak is quite wide in the τ domain (an order of magnitude, see Figure 5c) and, under the standard conditions (the optimal for the voltammetric peak, see Figure 2), the DRT peak is prominent, and the most significant effect is presented in the resistance with increasing KCl concentration. Accordingly, the main contributor to this peak is the IT event at the sample–membrane interface.

Peak I was related to the fastest process in the system and may be ascribed to the RC constant of the cell. Then, peak II manifested as a shoulder of peak I under some of the experimental conditions, being slightly more evident at higher potentials. Notably, the trends for τ and DRT resistances for this peak were not clear enough to draw trustworthy conclusions. For peak I, on one hand, there is an Ohmic resistance related to the bulk solution and the membrane resistance (*R*_b_), and it depends inversely on the conductivity and membrane dimensions (area and thickness). On the other hand, the geometric capacitance depends on the membrane dimensions but also the dielectric constant. In such cases, τ is known to be inversely proportional to the specific membrane conductivity (τ = ε/σ)^57^ and, hence, a cell constant of about 8.14 μs was determined: this value remains almost constant under all the experimental conditions that were assayed. Moreover, peak I presented a τ rather independent of *E*_dc_ under all the experimental conditions, whereas the DRT resistance varied as the following described.

At the standard experimental conditions (10 mM KCl, 40 mmol kg^–1^ NaTFPB and 17.8 mC cm^–2^ for the POT), the resistance was initially constant, then increasing with *E*_dc_, and finally decreasing at the highest *E*_dc_. Changing the electrolyte concentration to 100 mM made this trend more pronounced, showing a value of *E*_dc,min_ rather coincident with the *E*_peak_. Overall, an increase in the electrolyte concentration caused a significant decrease in the resistance and a relationship with *E*_dc_: this is the only peak showing a clear dependence on the electrolyte concentration in terms of DRT resistance, confirming the initial ascription. Regarding NaTFPB, inconclusive results were observed with the 80 mmol kg^–1^ concentration, while very similar results (V-shaped curve with the same *E*_dc,min_) were displayed for 120 and 160 mmol kg^–1^. Then, a minimum DRT resistance is achieved from a certain membrane conductivity in relation with the NaTFPB concentration. Finally, a similar trend but within slightly different resistance magnitude windows was presented under the three POT charge conditions.

Among all of the conclusions drawn from the DRT results, those related to the POT doping process are particularly relevant. Previous works attempting to describe (and theorize) the working mechanism of voltammetric ISEs based on POT assumed that the POT doping/undoping upon polarization has a neglected influence in the distribution of the applied potential and also in the generated current.^33^ This premise was grounded in the thin-layer behavior of the membrane. In essence, the doping/undoping process is not limited by mass transport within the membrane phase, and capacitor behavior occurs instead. It can be formulated that this behavior is due to the gradual absence of a net transport of charge at the POT–membrane interface, specifically due to steric hindrance of the entrance of TFPB^–^ into the POT lattice. In our system, this is reflected in an increase in the differential capacitance of peaks V and VI as the TFPB^–^ concentration in the membranes increased (Table 2). Notably, this effect is known as the “blocking wall boundary condition”,^58^ in relation to the absence of a net transport of charge through the interface, and instead, a capacitor is built.

**Figure 7:**
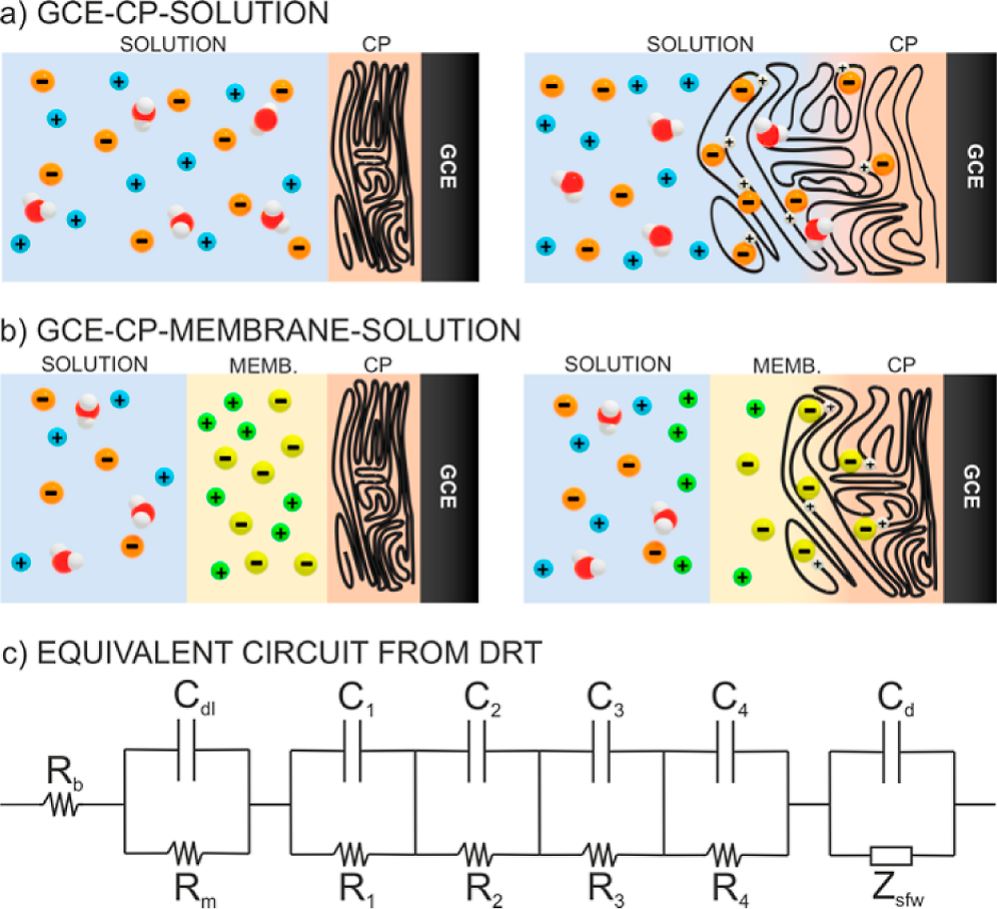
Schematic representations of: (a) the variation of volume in a GCE–CP–solution. (b) Variation of volume in a GCE–CP–membrane–solution. Left: before polarization. Right: after polarization. (c) Equivalent circuit of the GCE–POT–membrane–solution system. GCE: glassy carbon electrode.

It has been established that, in general, the oxidation of a CP in solution implies a volume expansion linked to a conformational relaxation that causes the polymer to swell, as illustrated in Figure 7a. This swelling allows the insertion of hydrophobic anions into the polymer lattice to dope the positive charges generated within the CP film. However, in our system, because the POT is physically confined between the membrane and electrode (GCE), the expansion is not freely allowed, and thus the insertion of the anion coming from the membrane is restricted, resembling the known blocking wall boundary condition (Figure 7b). Accordingly, the anion insertion rate is expected to decrease along a positive polarization event causing that, from a certain point in time (or potential), the POT–membrane interface becomes blocked, and a capacitor-like behavior is revealed. Moreover, the two different relaxation processes found in connection to the POT doping (i.e., peaks V and VI) likely relate to different conformations of the POT chains that involve different average rates of anion insertion in connection to an increasing restriction for the polymer expansion.

Figure 7c shows a simplified version of the outcomes from the DRT analysis expressed in terms of equivalent circuits. This is used herein to illustrate that DRT is much more convenient to treat complex systems, as we only consider the relaxation times to extract more detailed information. If DRT had not been used here, one may have directly set out the equivalent circuit shown in Figure 7c, which is not practical, to accurately treat the EIS data. This consists of a series of *RC* circuits with a finite length diffusion and is divided in turn into four elements that are connected in series. The first element corresponds to the Ohmic resistance (*R*_b_). The second element relates to the charge transfer across the membrane resistance and the capacitance of the double layer formed at the water–membrane interface (*R*_m_ and *C*_dl_). The third element corresponds to a series of *RC* circuits that resembles the different charge-transfer processes, including first the ion transfer at the membrane–solution interface and then the POT doping as deconvolved by the DRT method (*C*_1–4_ and *R*_1–4_). The fourth element describes the finite length diffusion (*C*_d_ and *Z*_sfw_), which explains the capacitor-like behavior for the mass transport within the membrane domain. Thus, DRT allows us to design an equivalent circuit based on the number of peaks that it resolves.

## Conclusions

The EIS–DRT tandem makes it possible to characterize the different processes involved in an electrochemical sensor based on a complex architecture of interconnected charge-transfer processes, such as solid-state voltammetric ISEs. A maximum of six events related to six different DRT peaks at medium–high frequencies were discerned. The fastest process corresponds to the *RC* constant of the cell. Next are the charge-transfer processes along the membrane phase and related to the membrane–solution interface. Finally, the slowest processes are ascribed to two different conformations of the oxidized CP, mainly related to the doping process, with anions coming from the membrane. These two peaks change with the applied *E*_dc_ potential following Butler–Volmer kinetics, which allows us to estimate the charge transfer related to the processes (0.8 mol of electrons) and the apparent rate constant *k*^0′^. EIS measurements showed that the overall process in the polarized ISE is not limited by diffusion in any of the faces but is limited by the formation of a capacitor at the POT–membrane interface. Indeed, the interface resembles the blocking wall boundary condition, showing the restriction of the CP swelling along the oxidation process and hence inhibiting the doping process (insertion of anions from the membrane into the CP lattice). DRT was also found to be useful to dig more deeply into the series interfaces than is achieved in traditional EIS-based conclusions. Opportunities to design optimal electrochemical sensors by identifying the limiting steps and their respective time scales are hereby opened. Furthermore, EIS–DRT may be applied to analogous systems comprising electrified series interfaces, such as those used in catalysts, batteries, and photovoltaic cells.

## Supporting Information Available

Example of DRT calculations; DRT parameters; EIS at different experimental conditions; plots of the relaxation times for the six DRT peaks; Q–Q plots; and additional chronoamperometry experiments (PDF)

## References

[ref1] Tarascon J.-M.; Armand M. Issues and Challenges Facing Rechargeable Lithium Batteries. Nature 2001, 414 (6861), 359–367. 10.1038/35104644. 11713543

[ref2] Von Hauff E. Impedance Spectroscopy for Emerging Photovoltaics. J. Phys. Chem. C 2019, 123 (18), 11329–11346. 10.1021/acs.jpcc.9b00892.

[ref3] Garcia-Belmonte G.; Guerrero A.; Bisquert J. Elucidating Operating Modes of Bulk-Heterojunction Solar Cells from Impedance Spectroscopy Analysis. J. Phys. Chem. Lett. 2013, 4 (6), 877–886. 10.1021/jz302064z. 26291350

[ref4] Bard A. J.; Faulkner L. R. Electrochemical Methods: Fundamentals and Applications, 2nd ed.; Wiley: New York, 2001.

[ref5] Pletcher D.; Greff R.; Peat R.; Peter L. M.; Robinson J. Instrumental Methods in Electrochemistry; Ellis Horwood Series in Physical Chemistry; Horwood: Chichester, 2011.

[ref6] Peter L. M. Dynamic Aspects of Semiconductor Photoelectrochemistry. Chem. Rev. 1990, 90 (5), 753–769. 10.1021/cr00103a005.

[ref7] Macdonald J. R.; Johnson W. B. Fundamentals of Impedance Spectroscopy. In Impedance Spectroscopy; Barsoukov E., Macdonald J. R., Eds.; John Wiley & Sons, Inc.: Hoboken, NJ, USA, 2018; pp 1–20. 10.1002/9781119381860.ch1.

[ref8] Han L.; Koide N.; Chiba Y.; Mitate T. Modeling of an Equivalent Circuit for Dye-Sensitized Solar Cells. Appl. Phys. Lett. 2004, 84 (13), 2433–2435. 10.1063/1.1690495.

[ref9] Ciucci F. Modeling Electrochemical Impedance Spectroscopy. Curr. Opin. Electrochem. 2019, 13, 132–139. 10.1016/j.coelec.2018.12.003.

[ref10] Macdonald J. R. Impedance Spectroscopy: Old Problems and New Developments. Electrochim. Acta 1990, 35 (10), 1483–1492. 10.1016/0013-4686(90)80002-6.

[ref11] Bazant M. Z.; Thornton K.; Ajdari A. Diffuse-Charge Dynamics in Electrochemical Systems. Phys. Rev. E 2004, 70 (2), 021506 10.1103/PhysRevE.70.021506. 15447495

[ref12] Schmidt J. P.; Berg P.; Schönleber M.; Weber A.; Ivers-Tiffée E. The Distribution of Relaxation Times as Basis for Generalized Time-Domain Models for Li-Ion Batteries. J. Power Sources 2013, 221, 70–77. 10.1016/j.jpowsour.2012.07.100.

[ref13] Dion F.; Lasia A. The Use of Regularization Methods in the Deconvolution of Underlying Distributions in Electrochemical Processes. J. Electroanal. Chem. 1999, 475 (1), 28–37. 10.1016/S0022-0728(99)00334-4.

[ref14] Dierickx S.; Weber A.; Ivers-Tiffée E. How the Distribution of Relaxation Times Enhances Complex Equivalent Circuit Models for Fuel Cells. Electrochim. Acta 2020, 355, 136764 10.1016/j.electacta.2020.136764.

[ref15] Meyer Q.; Liu S.; Li Y.; Zhao C. Operando Detection of Oxygen Reduction Reaction Kinetics of Fe-N-C Catalysts in Proton Exchange Membrane Fuel Cells. J. Power Sources 2022, 533, 231058 10.1016/j.jpowsour.2022.231058.

[ref16] Giesbrecht P. K.; Freund M. S. Investigation of Hydrogen Oxidation and Evolution Reactions at Porous Pt/C Electrodes in Nafion-Based Membrane Electrode Assemblies Using Impedance Spectroscopy and Distribution of Relaxation Times Analysis. J. Phys. Chem. C 2022, 126 (1), 132–150. 10.1021/acs.jpcc.1c09531.

[ref17] Naskar P.; Maiti A.; Chakraborty P.; Kundu D.; Biswas B.; Banerjee A. Chemical Supercapacitors: A Review Focusing on Metallic Compounds and Conducting Polymers. J. Mater. Chem. A 2021, 9 (4), 1970–2017. 10.1039/D0TA09655E.

[ref18] Ibanez J. G.; Rincón M. E.; Gutierrez-Granados S.; Chahma M.; Jaramillo-Quintero O. A.; Frontana-Uribe B. A. Conducting Polymers in the Fields of Energy, Environmental Remediation, and Chemical-Chiral Sensors. Chem. Rev. 2018, 118 (9), 4731–4816. 10.1021/acs.chemrev.7b00482. 29630346

[ref19] Puiggalí-Jou A.; del Valle L. J.; Alemán C. Drug Delivery Systems Based on Intrinsically Conducting Polymers. J. Controlled Release 2019, 309, 244–264. 10.1016/j.jconrel.2019.07.035. 31351927

[ref20] Bobacka J. Conducting Polymer-Based Solid-State Ion-Selective Electrodes. Electroanalysis 2006, 18 (1), 7–18. 10.1002/elan.200503384.

[ref21] Bobacka J.; Grzeszczuk M.; Ivaska A. Electrochemical Study of Poly(3-Octylthiophene) Film Electrodes. Impedance of the Polymer Film Semiconductor-Electrolyte Interface. Electrochim. Acta 1992, 37 (10), 1759–1765. 10.1016/0013-4686(92)85078-Y.

[ref22] Grzeszczuk M.; Bobacka J.; Ivaska A. Ion Transfer at a Poly(3-Octylthiophene) Film Electrode. J. Electroanal. Chem. 1993, 362 (1–2), 287–289. 10.1016/0022-0728(93)80032-D.

[ref23] Bobacka J.; Lindfors T.; McCarrick M.; Ivaska A.; Lewenstam A. Single-Piece All-Solid-State Ion-Selective Electrode. Anal. Chem. 1995, 67 (20), 3819–3823. 10.1021/ac00116a034.

[ref24] Bobacka J.; McCarrick M.; Lewenstam A.; Ivaska A. All Solid-State Poly(Vinyl Chloride) Membrane Ion-Selective Electrodes with Poly(3-Octylthiophene) Solid Internal Contact. Analyst 1994, 119 (9), 1985 10.1039/an9941901985.

[ref25] Michalska A.; Hulanicki A.; Lewenstam A. All Solid-State Hydrogen Ion-Selective Electrode Based on a Conducting Poly(Pyrrole) Solid Contact. Analyst 1994, 119 (11), 2417 10.1039/an9941902417.

[ref26] Crespo G. A.; Bakker E. Dynamic Electrochemistry with Ionophore Based Ion-Selective Membranes. RSC Adv. 2013, 3 (48), 25461 10.1039/c3ra43751e.

[ref27] Bobacka J.; Ivaska A.; Lewenstam A. Potentiometric Ion Sensors. Chem. Rev. 2008, 108 (2), 329–351. 10.1021/cr068100w. 18189426

[ref28] Hu J.; Stein A.; Bühlmann P. Rational Design of All-Solid-State Ion-Selective Electrodes and Reference Electrodes. TrAC Trends Anal. Chem. 2016, 76, 102–114. 10.1016/j.trac.2015.11.004.

[ref29] Liu Y.; Crespo G. A.; Cuartero M. Spectroelectrochemistry with Ultrathin Ion-Selective Membranes: Three Distinct Ranges for Analytical Sensing. Anal. Chem. 2022, 94 (25), 9140–9148. 10.1021/acs.analchem.2c01584. 35687727 PMC9244873

[ref30] Cuartero M.; Crespo G. A.; Bakker E. Ionophore-Based Voltammetric Ion Activity Sensing with Thin Layer Membranes. Anal. Chem. 2016, 88 (3), 1654–1660. 10.1021/acs.analchem.5b03611. 26712342

[ref31] Xu K.; Crespo G. A.; Cuartero M. Subnanomolar Detection of Ions Using Thin Voltammetric Membranes with Reduced Exchange Capacity. Sens. Actuators, B 2020, 321, 128453 10.1016/j.snb.2020.128453.

[ref32] Langmaier J.; Stejskalová K.; Samec Z. Evaluation of the Standard Ion Transfer Potentials for PVC Plasticized Membranes from Voltammetric Measurements. J. Electroanal. Chem. 2001, 496 (1–2), 143–147. 10.1016/S0022-0728(00)00337-5.

[ref33] Liu Y.; Crespo G. A.; Cuartero M. Semi-Empirical Treatment of Ionophore-Assisted Ion-Transfers in Ultrathin Membranes Coupled to a Redox Conducting Polymer. Electrochim. Acta 2021, 388, 138634 10.1016/j.electacta.2021.138634.

[ref34] Yuan D.; Cuartero M.; Crespo G. A.; Bakker E. Voltammetric Thin-Layer Ionophore-Based Films: Part 1. Experimental Evidence and Numerical Simulations. Anal. Chem. 2017, 89 (1), 586–594. 10.1021/acs.analchem.6b03354. 27976858

[ref35] Shi C.; Anson F. C. A Simple Method for Examining the Electrochemistry of Metalloporphyrins and Other Hydrophobic Reactants in Thin Layers of Organic Solvents Interposed between Graphite Electrodes and Aqueous Solutions. Anal. Chem. 1998, 70 (15), 3114–3118. 10.1021/ac980426k. 21644651

[ref36] Cuartero M.; Acres R. G.; De Marco R.; Bakker E.; Crespo G. A. Electrochemical Ion Transfer with Thin Films of Poly(3-Octylthiophene). Anal. Chem. 2016, 88 (13), 6939–6946. 10.1021/acs.analchem.6b01800. 27266678

[ref37] Cuartero M.; Crespo G. A.; Bakker E. Polyurethane Ionophore-Based Thin Layer Membranes for Voltammetric Ion Activity Sensing. Anal. Chem. 2016, 88 (11), 5649–5654. 10.1021/acs.analchem.6b01085. 27187779

[ref38] Lopes V. V.; Rangel C. M.; Novais A. Q. Modelling and Identification of the Dominant Phenomena in Hydrogen Fuel-Cells by the Application of DRT Analysis. Comput.-Aided Chem. Eng. 2013, 32, 283–288. 10.1016/B978-0-444-63234-0.50048-8.

[ref39] Schichlein H.; Müller A.; Voigts M.; Krügel A.; Ivers-Tiffée E. Deconvolution of Electrochemical Impedance Spectra for the Identification of Electrode Reaction Mechanisms in Solid Oxide Fuel Cells. J. Appl. Electrochem. 2002, 32 (8), 875–882. 10.1023/A:1020599525160.

[ref40] Wan T. H.; Saccoccio M.; Chen C.; Ciucci F. Influence of the Discretization Methods on the Distribution of Relaxation Times Deconvolution: Implementing Radial Basis Functions with DRTtools. Electrochim. Acta 2015, 184, 483–499. 10.1016/j.electacta.2015.09.097.

[ref41] Morgan F. D.; Lesmes D. P. Inversion for Dielectric Relaxation Spectra. J. Chem. Phys. 1994, 100 (1), 671–681. 10.1063/1.466932.

[ref42] R Core team. R: A Language and Environment for Statistical Computing, 2021.

[ref43] Liu Y.; Wiorek A.; Crespo G. A.; Cuartero M. Spectroelectrochemical Evidence of Interconnected Charge and Ion Transfer in Ultrathin Membranes Modulated by a Redox Conducting Polymer. Anal. Chem. 2020, 92 (20), 14085–14093. 10.1021/acs.analchem.0c03124. 32972129 PMC7584340

[ref44] Soliman H. M. A.; Kashyout A.-H. B. Electrochemical Deposition and Optimization of Thermoelectric Nanostructured Bismuth Telluride Thick Films. Engineering 2011, 03 (06), 659–667. 10.4236/eng.2011.36079.

[ref45] Timuda G. E.; Waki K. Galvanostatic Electrodeposition of ZnO Nanosheet: Effect of Different Applied Current Densities and Deposition Times on the Nanosheet Morphology. Adv. Nat. Sci. Nanosci. Nanotechnol. 2020, 11 (2), 025005 10.1088/2043-6254/ab7c60.

[ref46] Otero T. F.; Santos F. Polythiophene Oxidation: Rate Coefficients, Activation Energy and Conformational Energies. Electrochim. Acta 2008, 53 (7), 3166–3174. 10.1016/j.electacta.2007.10.072.

[ref47] Tanguy J. Modelization of the Electrochemical Behaviour of Conducting Polymers. Synth. Met. 1991, 43 (1–2), 2991–2994. 10.1016/0379-6779(91)91223-W.

[ref48] Bisquert J.; Garcia-Belmonte G.; Bueno P.; Longo E.; Bulhões L. Impedance of Constant Phase Element (CPE)-Blocked Diffusion in Film Electrodes. J. Electroanal. Chem. 1998, 452 (2), 229–234. 10.1016/S0022-0728(98)00115-6.

[ref49] Song J.; Bazant M. Z. Electrochemical Impedance Imaging via the Distribution of Diffusion Times. Phys. Rev. Lett. 2018, 120 (11), 116001 10.1103/PhysRevLett.120.116001. 29601735

[ref50] Bisquert J. Theory of the Impedance of Electron Diffusion and Recombination in a Thin Layer. J. Phys. Chem. B 2002, 106 (2), 325–333. 10.1021/jp011941g.

[ref51] Mei B.-A.; Munteshari O.; Lau J.; Dunn B.; Pilon L. Physical Interpretations of Nyquist Plots for EDLC Electrodes and Devices. J. Phys. Chem. C 2018, 122 (1), 194–206. 10.1021/acs.jpcc.7b10582.

[ref52] Otero T. F.; Romero M. C. Poly (3, 4-Ethylendioxithiophene) (PEDOT) Oxidation: Activation Energy and Conformational Energy. J. Phys.: Conf. Ser. 2008, 127, 012016 10.1088/1742-6596/127/1/012016.

[ref53] Yuan D.; Cuartero M.; Crespo G. A.; Bakker E. Voltammetric Thin-Layer Ionophore-Based Films: Part 2. Semi-Empirical Treatment. Anal. Chem. 2017, 89 (1), 595–602. 10.1021/acs.analchem.6b03355. 27976860

[ref54] Otero T. F.; Boyano I. Comparative Study of Conducting Polymers by the ESCR Model. J. Phys. Chem. B 2003, 107 (28), 6730–6738. 10.1021/jp027748j.

[ref55] Otero T. F.; Grande H.; Rodríguez J. Role of Conformational Relaxation on the Voltammetric Behavior of Polypyrrole. Experiments and Mathematical Model. J. Phys. Chem. B 1997, 101 (42), 8525–8533. 10.1021/jp9714633.

[ref56] Benz R.; Stark G.; Janko K.; Läuger P. Valinomycin-Mediated Ion Transport through Neutral Lipid Membranes: Influence of Hydrocarbon Chain Length and Temperature. J. Membr. Biol. 1973, 14, 339–364. 10.1007/BF01868084. 4781449

[ref57] Horvai G.; Graf E.; Toth K.; Pungor E.; Buck R. P. Plasticized Poly(Vinyl Chloride) Properties and Characteristics of Valinomycin Electrodes. 1. High-Frequency Resistances and Dielectric Properties. Anal. Chem. 1986, 58 (13), 2735–2740. 10.1021/ac00126a034.

[ref58] Cooper S. J.; Bertei A.; Finegan D. P.; Brandon N. P. Simulated Impedance of Diffusion in Porous Media. Electrochim. Acta 2017, 251, 681–689. 10.1016/j.electacta.2017.07.152.

