# Supplementary material for: Usefulness of the Distribution of Relaxation Time Method in Electroanalytical Systems: The Case of Voltammetric Ion-Selective Electrodes
Source: ACS Omega. 2024 Feb 6;9(7):8162–72. doi: 10.1021/acsomega.3c08656 (PMC10882611; doi:10.1021/acsomega.3c08656)
Supplement: Supplementary file 1 — ao3c08656_si_001.pdf [file ao3c08656_si_001.pdf]

Supporting Information for:

Usefulness of the Distribution of Relaxation Times Method in Electroanalytical Systems: The Case of Voltammetric Ion-Selective Electrodes

Iván Robayo-Molina[a], Gastón A. Crespo[a,b,*] and María Cuartero[a,b,*]

[a]Deparment of Chemistry, School of Engineering Science in Chemistry, Biotechnology and Health, KTH Royal Institute of Technology, Teknikringen 30, SE-100 44, Stockholm, Sweden.

[b]UCAM-SENS, Universidad Católica San Antonio de Murcia, UCAM Hitech, Avda. Andres Hernandez Ros 1, 30107, Murcia Spain.

(*) Corresponding authors: gacp@kth.se; mariacb@kth.se

TABLE OF CONTENTS

		Page
**Exemplifying DRT calculation using the DRTtools**	....	S3
**Tables**	....	S4
Table S1. Numerical values for the elements in the exemplary circuit.	....	S4
Table S2. Results showcasing the DRT results from the impedance of the exemplary circuit.	....	S4
Table S3. Electrochemical parameters from Figure 2a-b	....	S4
Table S4. Estimation of [TFPB$^-$] amount consumed during oxidation of POT in thin-layer membranes (~200 nm l thickness)	....	S4
Table S5. Time scale of the peaks resolved by DRT. Electrolyte concentration.	....	S5
Table S6. Time scale of the peaks resolved by DRT. NaTFPB concentration.	....	S6
Table S7. Time scale of the peaks resolved by DRT. POT charge	....	S8
Table S8. Resistance of the peaks resolved by DRT. Electrolyte concentration.	....	S9
Table S9. Resistance of the peaks resolved by DRT. NaTFPB concentration.	....	S10
Table S10. Resistance of the peaks resolved by DRT. POT charge.	....	S12
**Figures**	....	S13
Figure S1. Nyquist plot derived from simulating the exemplary circuit.	....	S13
Figure S2. Corresponding Distribution of Relaxation Times of the exemplary circuit	....	S13
Figure S3. EIS as a function of the applied potential. Standard experimental conditions.	....	S14
Figure S4. Impedance spectra obtained using three electrodes and at different E_{dc} inputs, a) E_{peak} – 20 mV, b) E_{peak} and c) E_{peak} + 20mV.	....	S15
Figure S5. *K*ramers-Kronig Transform Test Results for Electrodes 1, 2, and 3, based on the data presented in Figure S13.	....	S16
Figure S6. EIS as a function of the applied potential. Varying the experimental conditions	....	S17
Figure S7. Imaginary part of the impedance as a function of $1/f$.	....	S18
Figure S8. Differential capacitance as a function of the TFPB$^-$.	....	S18
Figure S9. For Peak I, plot of the logarithm of the relaxation time values (left) and resistanc (right) as a function of the Edc.	....	S19
Figure S10. For Peak II, plot of the logarithm of the relaxation time values (left) and resistance (right) as a function of the Edc.	....	S20
Figure S11. For Peak III, plot of the logarithm of the relaxation time values (left) and resistance (right) as a function of the Edc.	....	S21
Figure S12. For Peak IV, plot of the logarithm of the relaxation time values (left) and resistance (right) as a function of the Edc.	....	S22
Figure S13. For Peak V, plot of the logarithm of the relaxation time values (left) and resistance (right) as a function of the Edc.	....	S23
Figure S14. Quantile-Quantile plot for peak V at different NaTFPB concentrations.	....	S24
Figure S15. Quantile-Quantile plot for peak VI at different NaTFPB concentrations.	....	S24
Figure S16. Chronoamperometry.	....	S25
**References**	....	S26

Exemplifying DRT calculation using the *DRTtools*

The DRT of the impedance data was calculated with a regularization parameter λ of 10^{-3}, using the Matlab® software with the package *DRTtools*. This package includes a graphical user interphase that is rather intuitive to handle. The EIS data is imported as a text file (.txt or .csv). This file must have three columns. The first column for the frequency data, the second one for the real part of the EIS data, and the third one for the imaginary parts. No labels for the columns must be included. More detailed information to operate *DRTtools* can be found in the link: https://sites.google.com/site/drttools/home.

Following, an easy example on how to operate is provided. The EIS of a circuit consisting of a resistor in series with two RC circuits was simulated. The frequency range selected for the simulation was from 1×10^{6} Hz to 0.01 Hz. The calculated Nyquist plot is shown in **Figure S1**. Two overlapped semi-circles appeared. The numerical values of the parameters together with the time constant for each RC component are shown in **Table S1**. The results from the DRT analysis are shown in **Figure S2** and **Table S2**. Two peaks were revealed. The first peak is associated with the first RC component of the circuit. The resistance calculated from the DRT is very close to the real resistance, with a relative deviation of 2.5%. The second peak is associated with the second RC component of the circuit The calculated resistance presents a relative deviation of 2.3% with respect to the real value. The main differences between the two sets of data are a consequence of the time scale, i.e., how the relaxation time (τ) was calculated. In EIS, τ was calculated as the product of R_2C_1, for each RC component of the circuit; whereas DRT calculates τ considering R_1 as well. The second RC component is calculated based on the previous R-RC equivalent circuit.

Tables

Table S1. Numerical values for the elements in the exemplary circuit.

R_1	100 Ω
R_2	200 Ω
C_1	1×10^{-5} F
R_3	300 Ω
C_2	7.5×10^{-5} F
τ_1	200 µs
τ_2	22.5 ms

Table S2. Results showcasing the DRT results from the impedance of the exemplary circuit.

Peak #	Area / Ω	tau
1	195	311 µs
2	307	3.48 ms

Table S3. Electrochemical parameters observed in the experiments presented in Figure 2.

POT /mC cm^{-2}	[TFPB$^-$] /mmol kg^{-1}	Q_{anodic} /µC	$Q_{cathodic}$ /µC	E_{anodic} /V	$E_{cathodic}$ /V
17.8	40	11.147	11.143	0.264	0.249
17.8	80	18.780	18.055	0.309	0.279
17.8	120	29.817	30.362	0.363	0.326
17.8	160	42.138	44.681	0.413	0.338
14.3	40	7.229	6.306	0.309	0.297
21.4	40	6.784	6.472	0.308	0.274

Observation: there is a rather good agreement between the charge related to the oxidation and reduction peaks for all the conditions, with <10% of deviation, except for the case containing the largest amount of TFBP$^-$, with a 25% deviation. For the optimized amount of POT (17.8 mC. cm^{-2}), the increase of TFPB$^-$ above 80 mmol.kg^{-1} resulted in a clear appearance of a second peak, which is likely associated to another doping process and accompanied by changes in the morphology of the film (e.g., expansion/contraction, pores, cracks, loss of reactivity, etc.).

Table S4. Estimation of the TFPB$^-$ amount that is consumed during the oxidation of POT in thin-layer membranes with thickness of 230 nm, density of 1 g cm^{-3} and assuming a diameter of 5 mm (equal to the POT surface).

TFPB in the membrane / mmol kg^{-1}	Membrane volume / cm^{-3}	Membrane mass / kg	mol of TFPB$^-$ in the membrane	Charge of TFPB$^-$ in the membrane / µC	Charge under the voltammetric peak / µC	% of consumed TFPB$^-$
40	4.52x10^{-6}	4.52x10^{-9}	1.81x10^{-10}	17.4	11.1	64.0

Table S5. Time scale of the peaks resolved by DRT. The time scale for each peak is shown as a function of the applied potential and the electrolyte concentration.

E_{app}/mV	τ_1/µs	τ_2/µs	τ_3/µs	τ_4/µs	τ_5/ms	τ_6/ms
**10 mM KCl**						
150	8.14	—	—	164.5	2.057	25.26
160	8.14	—	—	150.0	1.680	19.15
180	8.14	—	—	124.8	1.205	11.22
200	8.14	—	—	122.5	1.228	6.95
220	8.14	—	—	129.4	1.205	4.22
240	8.14	—	—	139.3	0.377	2.61
260	8.14	—	—	155.6	0.370	1.81
280	8.14	—	—	161.5	0.325	1.35
300	8.14	—	—	147.3	0.234	0.93
320	8.14	—	—	152.8	0.234	0.97
340	8.14	—	—	147.3	0.314	2.47
360	8.14	—	—	94.6	0.507	4.63
380	8.30	—	—	65.4	0.516	6.70
400	8.45	12.68	38.33	122.5	0.632	9.16
420	8.14	14.97	38.33	167.6	0.789	10.62
440	8.00	14.16	31.88	107.6	0.881	10.62
450	8.30	20.48	46.09	205.2	0.789	9.33
**100 mM KCl**						
300	2.01	—	19.03	275.7	3.142	37.88
320	2.01	—	18.34	187.2	1.983	22.61
340	2.01	—	18.00	131.9	1.346	14.00
360	2.01	—	18.00	105.7	1.022	8.83
380	2.01	—	22.46	105.7	0.836	5.67
400	2.01	—	20.11	79.2	0.719	3.78
420	2.01	—	19.74	190.7	0.598	2.76
440	1.93	2.96	18.34	74.5	0.266	1.95
460	1.93	2.80	8.61	27.5	0.229	1.45
480	1.97	3.12	8.61	50.6	0.229	1.83
500	1.97	3.07	7.03	87.9	0.535	3.64
520	1.97	3.69	8.45	90.8	0.644	6.82
540	1.93	4.43	9.80	122.5	0.733	9.50
560	1.93	5.74	12.68	144.6	0.865	11.86
580	1.93	5.74	14.17	152.8	1.021	13.74
600	2.01	8.93	19.38	194.2	1.205	17.46

Relative standard deviations (RSD) for τ were calculated by a triplicate experiment resulting in $\leq$16%

Table S6. Time scale of the peaks resolved by DRT. The time scale for each peak is shown as a function of the applied potential and the TFPB⁻ concentration in the membrane.

E_{app}/mV	τ_1/µs	τ_2/µs	τ_3/µs	τ_4/µs	τ_5/ms	τ_6/ms
**80 mmol TFPB^{-1} kg^{-1} of membrane**						
100	5.11	8.41	31.17	330.4	3.438	42.24
120	5.11	8.41	31.75	260.0	2.607	28.15
140	5.11	8.41	33.56	173.3	1.644	15.89
160	5.11	7.96	40.36	133.8	1.116	9.48
180	5.11	—	37.49	78.4	0.678	5.35
200	5.11	—	36.13	68.9	0.461	3.08
220	5.11	—	35.47	57.3	0.383	1.74
240	5.11	—	—	48.5	0.113	0.79
260	5.11	—	—	50.4	0.083	0.46
280	5.11	—	—	66.4	—	0.44
300	5.11	—	—	109.3	—	0.53
320	5.11	—	—	183.1	—	1.04
340	5.11	—	—	183.1	—	1.12
360	5.11	—	—	122.0	—	1.20
380	5.11	—	—	61.7	—	1.20
400	5.11	—	—	39.6	0.296	1.61
420	5.11	8.11	21.56	35.5	0.330	2.56
440	5.11	7.13	21.56	45.9	0.383	3.38
460	5.11	7.53	20.77	49.4	0.666	5.55
480	5.11	7.82	25.92	71.5	0.895	7.74
500	4.93	7.00	29.49	96.0	1.096	8.97
520	5.11	8.89	34.18	131.4	1.270	10.40
**120 mmol TFPB^{-1} kg^{-1} of membrane**						
150	8.14	—	—	120.2	1.322	15.07
160	8.14	—	—	107.6	1.099	12.08
180	8.14	—	—	87.9	0.897	7.34
200	8.14	—	—	86.3	0.760	4.22
220	8.14	—	—	111.7	0.644	2.57
240	8.14	—	—	107.6	0.566	1.71
260	8.14	—	—	98.2	0.217	1.02
280	8.14	—	—	81.6	0.184	0.62
300	8.14	—	—	80.1	0.191	0.42
320	8.14	—	—	96.4	—	0.23
340	8.14	—	—	131.8	—	—
360	8.14	—	—	147.3	—	—
380	8.14	—	—	183.7	—	—
400	8.14	—	—	216.9	—	—
420	8.14	—	—	221.0	0.357	0.68
440	8.14	—	—	216.9	0.325	0.93

460	8.14	—	—	213.0	—	1.78
480	8.14	—	—	183.7	—	2.57
500	8.14	—	—	155.6	—	3.03
520	8.14	—	—	120.2	0.429	4.46
540	8.14	—	—	100.0	0.471	5.08
560	8.14	—	—	81.6	0.507	5.88
580	8.14	—	—	80.1	0.488	5.57
600	8.14	—	—	77.2	0.576	6.82
**160 mmol TFPB^{-1} kg^{-1} of membrane**						
150	8.14	—	—	100.0	0.914	7.08
160	8.14	—	—	89.5	0.914	5.08
180	8.14	—	—	94.6	0.746	2.76
200	8.14	—	—	113.8	0.598	1.62
220	8.14	—	—	109.6	0.350	0.95
240	8.14	—	—	81.6	0.213	0.49
260	8.14	—	—	84.7	0.153	0.27
280	8.14	—	—	—	0.137	—
300	8.14	—	—	—	0.110	—
320	8.14	—	—	—	0.106	—
340	8.14	—	—	—	—	—
360	8.14	—	—	—	—	—
380	8.14	—	—	—	—	—
400	8.14	—	—	—	—	—
420	8.14	—	—	213.0	—	—
440	8.14	—	—	180.4	—	—
460	8.14	—	—	180.4	—	—
480	8.14	—	—	183.7	—	—
500	8.14	—	—	109.6	—	0.64
520	8.14	—	—	84.7	—	1.00
540	8.14	—	—	71.7	—	1.37
560	8.14	—	—	65.4	0.818	1.91
580	8.14	—	—	63.1	0.488	3.09
600	8.14	—	—	63.1	0.526	4.38
620	8.14	—	—	66.6	0.545	5.67
640	8.14	—	—	70.4	0.566	6.57
660	8.14	—	—	77.2	0.609	6.95

% Relative standard deviations (RSD) for τ were estimated to be ≤16%

Table S7. Time scale of the peaks resolved by DRT. The time scale for each peak is shown as a function of the applied potential and the charged of the synthetized POT.

E_{app}/mV	τ_1/µs	τ_2/µs	τ_3/µs	τ_4/µs	τ_5/ms	τ_6/ms
			21.4 mC cm^{-2}			
200	5.01	—	52.1	341.4	3.483	53.29
220	5.01	—	55.06	268.7	2.546	34.24
240	5.01	—	68.69	192.8	1.967	23.68
260	5.01	—	61.5	149.0	1.492	16.38
280	5.01	—	61.5	113.0	1.218	11.97
300	5.01	—	63.81	117.2	1.174	9.60
320	5.01	—	71.27	133.4	1.360	7.98
340	5.01	—	59.27	172.6	1.931	8.75
360	5.01	—	63.81	231.8	2.365	10.72
380	5.01	7.95	57.13	215.4	2.117	14.40
400	5.01	7.95	47.51	203.8	2.041	17.00
420	5.01	7.95	39.51	189.3	1.861	17.00
440	4.83	6.73	35.38	135.8	1.438	14.40
460	4.83	7.12	25.39	88.9	1.013	10.14
			14.3 mC cm^{-2}			
200	5.01	—	38.1	203.8	2.238	33.61
220	5.01	—	41.8	149.0	1.636	21.20
240	5.01	—	51.1	108.9	1.131	13.13
260	5.01	—	54.1	101.2	0.827	8.75
280	5.01	—	54.6	627.3	6.639	6.40
300	5.01	—	55.1	62.6	0.467	4.34
320	5.01	—	51.1	—	0.361	3.30
340	5.01	—	47.5	—	0.274	2.32
360	5.01	—	40.2	—	0.232	1.90
380	5.01	—	37.4	—	0.223	1.79
400	5.01	7.95	31.1	—	0.241	1.86
420	4.92	6.73	26.3	52.1	0.354	2.84
440	4.92	6.99	21.9	44.1	0.396	3.68
460	4.83	6.99	17.2	33.5	0.403	4.43
480	4.83	6.86	17.9	33.5	0.361	4.59
500	4.92	8.10	20.7	36.7	0.306	3.61

% Relative standard deviations (RSD) for τ were estimated to be ≤16%

Table S8. Resistance of the peaks resolved by DRT. The time scale for each peak is shown as a function of the applied potential and the electrolyte concentration.

E_{app}/mV	R_1/Ω	R_2/kΩ	R_3/kΩ	R_4/Ω	R_5/kΩ	R_6/kΩ
**10 mM KCl**						
150	264.2	—	—	224.3	1.153	17.31
160	267.4	—	—	197.0	0.970	13.55
180	266.0	—	—	170.3	0.779	7.608
200	268.2	—	—	149.3	0.701	4.044
220	269.5	—	—	120.7	0.682	1.959
240	284.5	—	—	118.5	0.215	1.248
260	317.9	—	—	130.9	0.182	0.617
280	364.4	—	—	117.9	0.145	0.311
300	404.6	—	—	65.2	0.110	0.192
320	441.7	—	—	55.3	0.110	0.113
340	459.9	—	—	118.3	0.098	0.085
360	493.0	—	—	153.4	0.157	0.132
380	520.5	0.390	—	273.1	0.245	0.289
400	559.5	0.384	0.413	79.8	0.428	0.577
420	594.3	0.279	0.629	217.4	0.626	0.887
440	598.5	0.189	1.489	318.0	1.187	1.280
450	547.8	0.231	1.395	591.7	1.531	1.301
**100 mM KCl**						
300	229.5	—	0.224	329.4	1.680	28.33
320	230.4	—	0.186	243.6	1.247	17.49
340	241.9	—	0.156	209.1	1.124	9.701
360	255.6	—	0.165	190.1	0.906	5.473
380	276.6	—	0.170	110.0	0.693	3.145
400	301.5	—	0.193	144.5	0.393	1.903
420	336.4	—	0.192	145.2	0.253	1.174
440	208.5	0.177	0.166	66.0	0.248	0.757
460	130.0	0.277	0.140	100.7	0.260	0.480
480	84.3	0.379	0.275	120.9	0.201	0.331
500	70.5	0.219	0.593	187.2	0.276	0.182
520	43.7	0.282	0.792	137.8	0.310	0.324
540	47.8	0.216	1.126	172.6	0.296	0.619
560	58.1	0.324	1.352	198.6	0.359	1.030
580	80.3	0.254	1.814	210.1	0.541	1.527
600	105.7	0.093	2.965	367.6	0.952	2.059

Relative standard deviations (RSD) for **R** were calculated by a triplicate experiment resulting in ≤15%

Table S9. Resistance of the peaks resolved by DRT. The time scale for each peak is shown as a function of the applied potential and the TFPB⁻ concentration in the membrane.

E_{app}/mV	R_1/Ω	R_2/Ω	R_3/Ω	R_4/Ω	R_5/kΩ	R_6/kΩ
**80 mmol TFPB^{-1} kg^{-1} of membrane**						
110	394.8	59.09	453.9	502.9	2.432	31.86
130	384.5	49.65	400.9	431.2	2.142	20.23
150	378.1	39.07	355.2	313.9	1.683	10.48
170	349.4	36.07	336.2	191.8	1.369	4.960
190	345.5	—	207.2	159.7	0.875	2.319
210	309.0	—	172.1	184.7	0.768	2.352
230	272.9	—	51.01	162.2	0.276	0.380
250	239.3	—	—	148.9	0.087	0.226
270	213.9	—	—	83.12	0.036	0.113
290	195.8	—	—	56.46	—	0.062
310	181.7	—	—	27.89	—	0.046
330	192.8	—	—	28.26	—	0.046
350	209.0	—	—	27.19	—	0.054
370	249.2	—	—	36.76	—	0.067
390	305.1	—	—	62.27	—	0.086
410	371.1	—	—	115.6	0.043	0.092
430	336.5	99.54	28.02	234.1	0.088	0.134
450	231.1	189.2	323.8	234.1	0.163	0.218
470	241.4	108.5	482.7	657.6	0.235	0.385
490	235.3	101.3	756.6	1115	0.412	0.680
510	188.3	120.3	843.5	2056	0.715	1.195
530	242.7	95.33	863.7	3008	1.129	1.829
**120 mmol TFPB^{-1} kg^{-1} of membrane**						
150	194.7	—	—	174.3	0.845	10.79
160	185.5	—	—	151.8	0.716	8.256
180	170.3	—	—	138.7	0.489	4.524
200	158.5	—	—	140.6	0.356	2.319
220	145.5	—	—	126.7	0.320	1.094
240	138.8	—	—	119.4	0.275	0.452
260	126.8	—	—	104.2	0.070	0.289
280	115.5	—	—	73.32	0.067	0.114
300	100.7	—	—	60.23	0.022	0.059
320	97.8	—	—	38.52	—	0.035
340	89.0	—	—	38.93	—	—
360	84.6	—	—	27.84	—	—
380	82.7	—	—	20.60	—	—
400	87.7	—	—	18.48	—	—
420	102.4	—	—	11.18	0.008	0.007
440	127.0	—	—	14.56	0.006	0.015

460	163.0	—	—	32.02	—	0.030
480	210.8	—	—	50.41	—	0.056
500	261.5	—	—	78.74	—	0.088
520	371.5	—	—	139.3	0.031	0.167
540	403.0	—	—	174.0	0.051	0.205
560	494.7	—	—	259.9	0.133	0.371
580	512.3	—	—	307.5	0.145	0.386
600	588.1	—	—	430.8	0.239	0.679
**160 mmol TFPB^{-1} kg^{-1} of membrane**						
150	263.6	—	—	165.7	0.588	3.851
160	258.3	—	—	166.0	0.554	2.629
180	238.1	—	—	165.0	0.464	1.186
200	216.6	—	—	170.9	0.311	0.488
220	196.4	—	—	122.6	0.124	0.235
240	179.0	—	—	74.29	0.074	0.107
260	164.8	—	—	28.09	0.063	0.035
280	141.5	—	—	—	0.067	—
300	125.8	—	—	—	0.024	—
320	112.0	—	—	—	0.005	—
340	98.4	—	—	—	—	—
360	89.2	—	—	—	—	—
380	83.9	—	—	—	—	—
400	83.1	—	—	—	—	—
420	101.1	—	—	6.00	—	—
440	105.0	—	—	6.27	—	—
460	119.7	—	—	12.50	—	—
480	143.6	—	—	24.11	—	—
500	175.9	—	—	31.53	—	0.017
520	214.9	—	—	60.26	—	0.030
540	256.7	—	—	109.0	—	0.049
560	296.6	—	—	180.5	0.022	0.053
580	334.3	—	—	279.7	0.046	0.097
600	369.5	—	—	418.9	0.089	0.158
620	424.8	—	—	636.4	0.140	0.263
640	449.3	—	—	795.9	0.267	0.382
660	472.5	—	—	1013	0.355	0.522

% Relative standard deviations (RSD) for **R** were estimated to be ≤15%

Table S10. Resistance of the peaks resolved by DRT. The time scale for each peak is shown as a function of the applied potential and the charged of the synthetized POT.

E_{app}/mV	R_1/Ω	R_2/Ω	R_3/Ω	R_4/Ω	R_5/kΩ	R_6/kΩ
			**21.4 mC cm^{-2}**			
200	347.2	—	188.7	337.9	1.848	46.144
220	34679.7	—	174.1	276.5	1.517	28.718
240	382.3	—	190.9	200.9	1.284	18.107
260	412.6	—	162.5	155.3	1.174	11.768
280	454.9	—	138.7	166.8	1.004	7.927
300	509.8	—	166.6	156.4	0.927	5.689
320	571.3	—	176.6	131.4	1.086	4.337
340	635.2	—	191.1	170.9	1.514	3.350
360	699.6	—	242.5	194.1	1.814	3.059
380	619.1	116.7	203.1	235.4	1.975	3.615
400	611.9	158.3	167.1	270.4	2.051	4.191
420	599.6	201.1	192.5	234.6	1.743	4.059
440	363.3	474.8	165.3	227.8	1.328	4.199
460	350.5	506.1	188.7	205.1	0.952	3.016
			**14.3 mC cm^{-2}**			
200	208.9	—	136.4	176.8	1.10	25.35
220	211.1	—	112.7	151.5	0.882	15.82
240	213.9	—	128.1	91.8	0.689	9.50
260	219.2	—	143.4	44.5	0.541	5.78
280	222.2	—	134.6	24.7	0.462	3.69
300	239.7	—	127.7	11.5	0.338	2.35
320	265.6	—	125.4	—	0.259	1.54
340	301.0	—	127.0	—	0.201	1.06
360	343.0	—	122.0	—	0.186	0.772
380	395.8	—	160.7	—	0.176	0.591
400	338.2	95.2	188.7	—	0.204	0.511
420	282.2	194.3	224.2	31.8	0.279	0.504
440	176.3	298.1	290.3	63.7	0.307	0.641
460	169.7	265.7	291.2	185.9	0.310	0.843
480	158.3	221.4	465.1	145.3	0.292	1.001
500	155.0	309.6	500.9	97.6	0.239	0.894

% Relative standard deviations (RSD) for **R** were estimated to be ≤15%

Figures

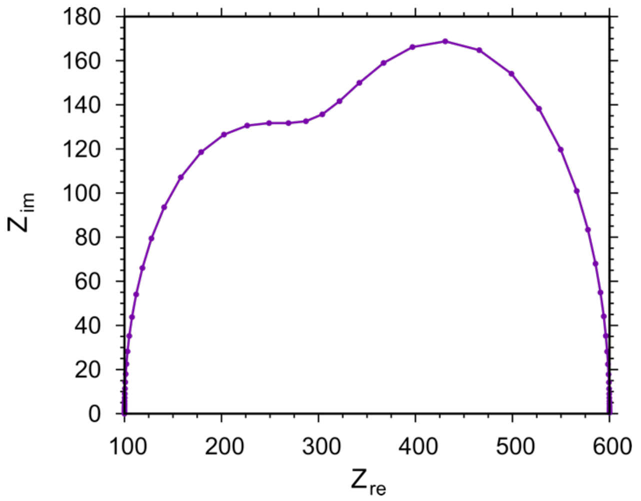

Figure S1. Nyquist plot derived from simulating the exemplary circuit.

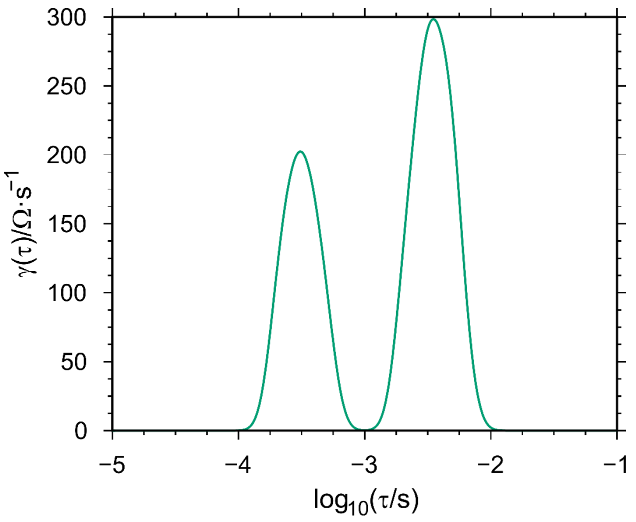

Figure S2. Corresponding Distribution of Relaxation Times of the exemplary circuit

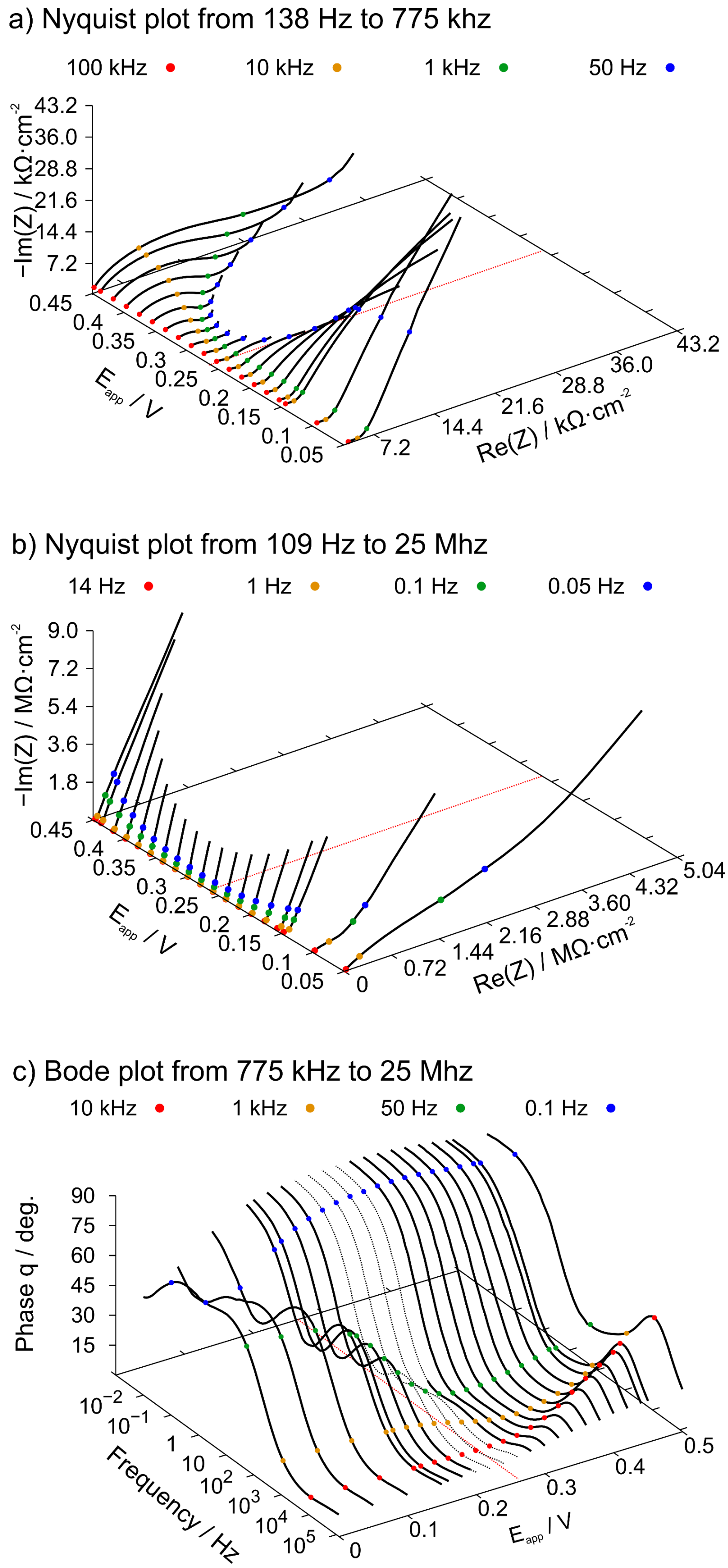

Figure S3. EIS as a function of the applied potential in a system consisting of a POT film of 17.8 mC cm^{-2}), a membrane with 40 mmol/kg^{-1} of TFPB^{-} and 10 mM KCl as the electrolyte: Nyquist plots at **(a)** high frequencies (138 Hz – 775 kHz) and **(b)** low frequencies (109 Hz – 25 mHz). **(c)** Bode plot. The red line indicates the E_{peak}.

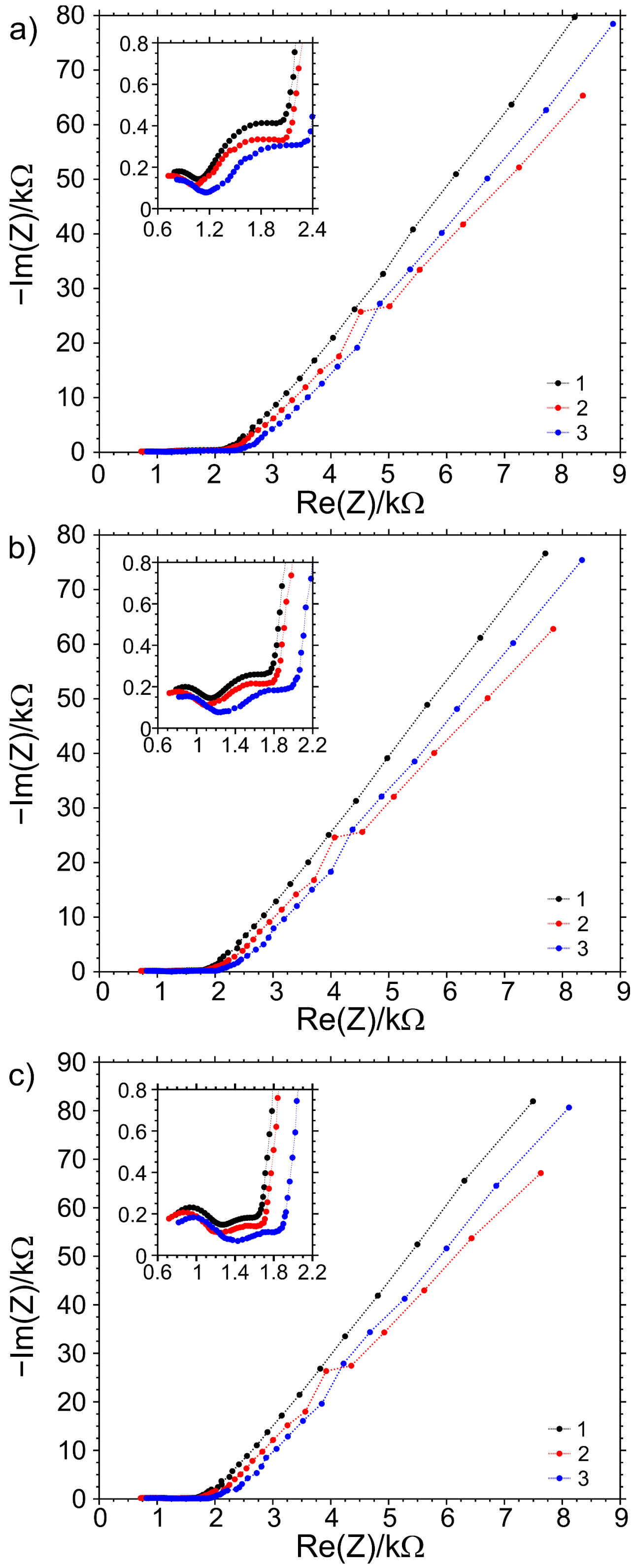

Figure S4. Impedance spectra obtained at different E_{dc} inputs, a) E_{peak} – 20 mV, b) E_{peak} and c) E_{peak} + 20mV. Three electrodes were analogously prepared: POT film (17.8 mC cm^{-2}) and the membrane containing 40 mmol kg^{-1} of NaTFPB. Background electrolyte: 10 mM KCl.

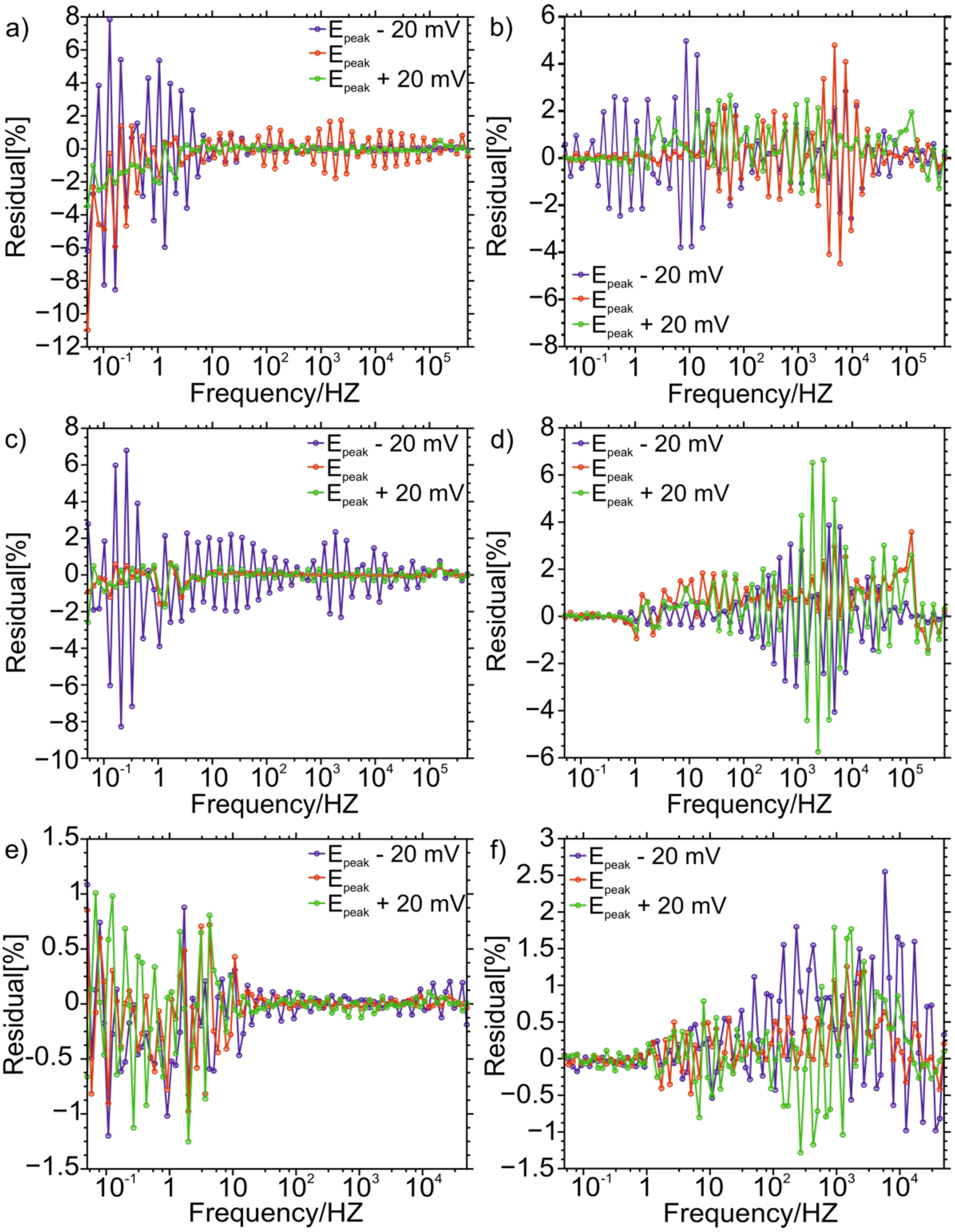

Figure S5. Kramers-Kronig Transform Test Results for the data shown in Figure S2. Panels (a) and (b) correspond to electrode 1, (c) and (d) to electrode 2, (e) and (f) to electrode 3. The right panels display the imaginary part, while the left panels present the real part of the impedance.

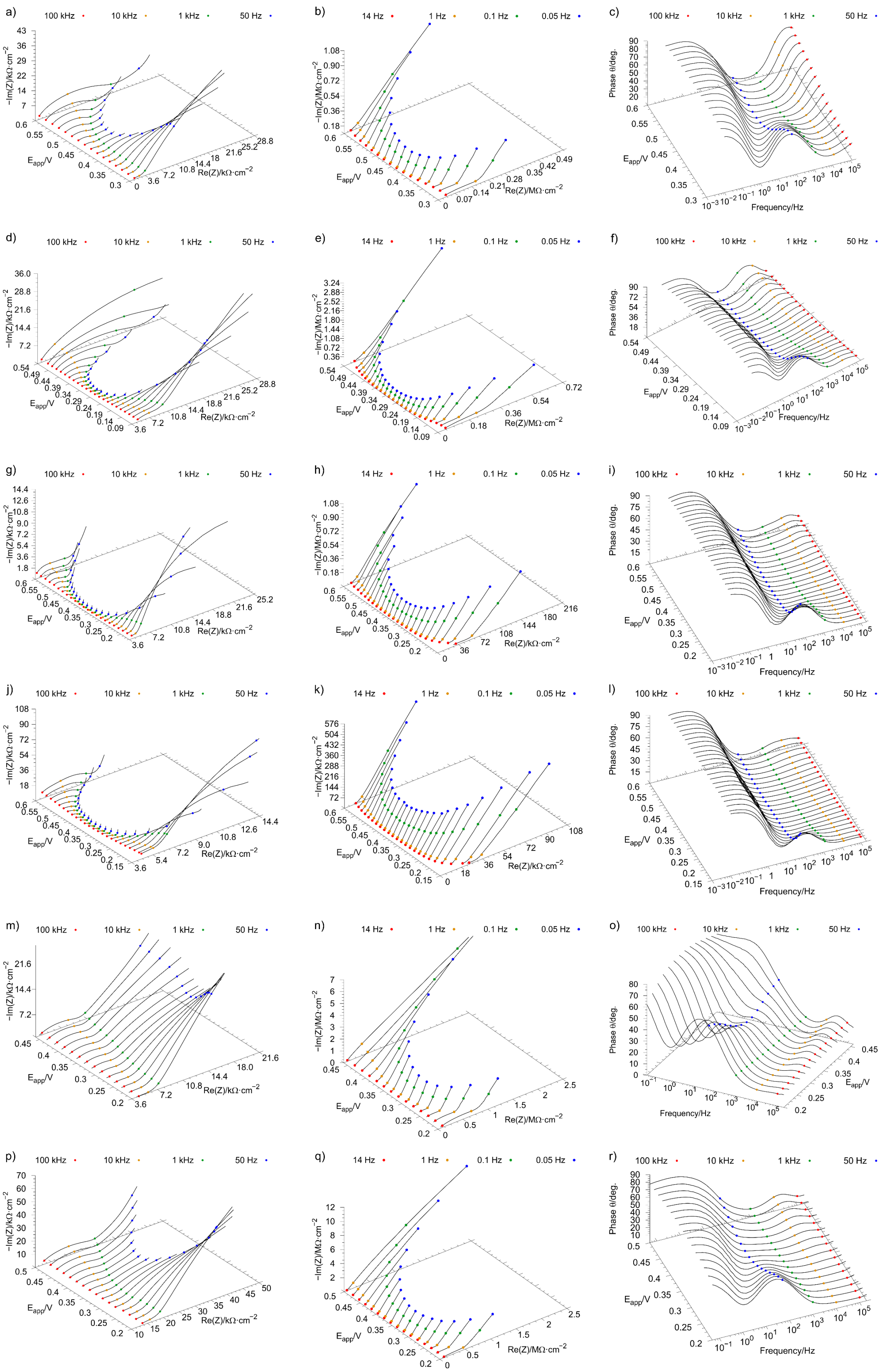

Figure S6. EIS as a function of the applied potential varying the electrolyte concentration, the TFPB⁻ concentration in the membrane and the charge of the POT film. Left: Nyquist plots at high frequencies (138 Hz – 250 kHz). Center: Nyquist plots at low frequencies (109 Hz – 25 mHz). Right: Bode plots**. (a-c)** 100 mM KCl; (d-f) 80 mmol kg^{-1}; **(g-i)** 120 mmol kg^{-1}; **(j-l)** 160 mmol kg^{-1}; **(m-o)** 21.4 mC cm^{-2}; and **(p-r)** 14.3 mC cm^{-2}.

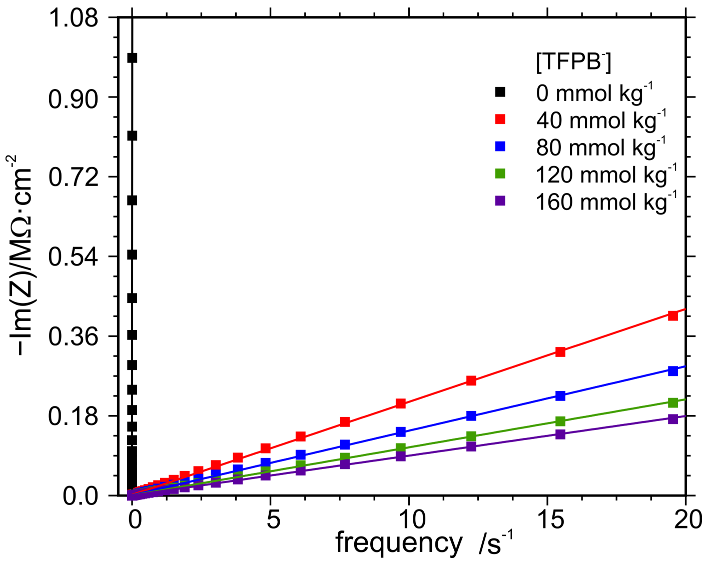

Figure S7. Imaginary part of the impedance $-Z_{im}$ as a function of $1/f$ for low frequencies.

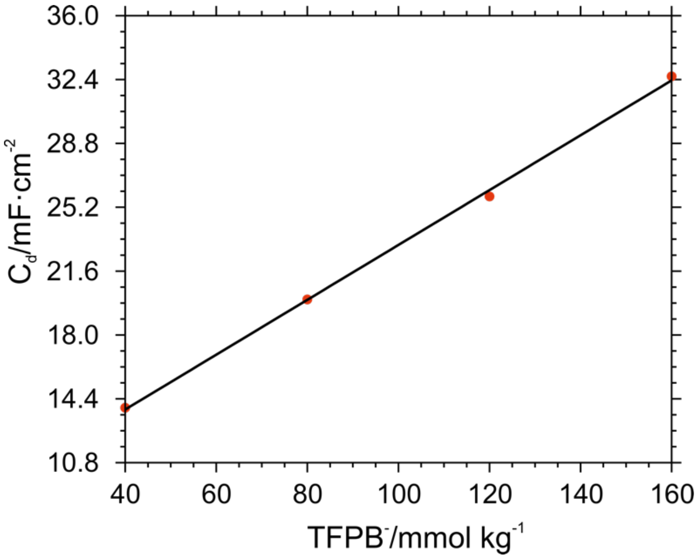

Figure S8. Differential capacitance as a function of the TFPB- concentration.

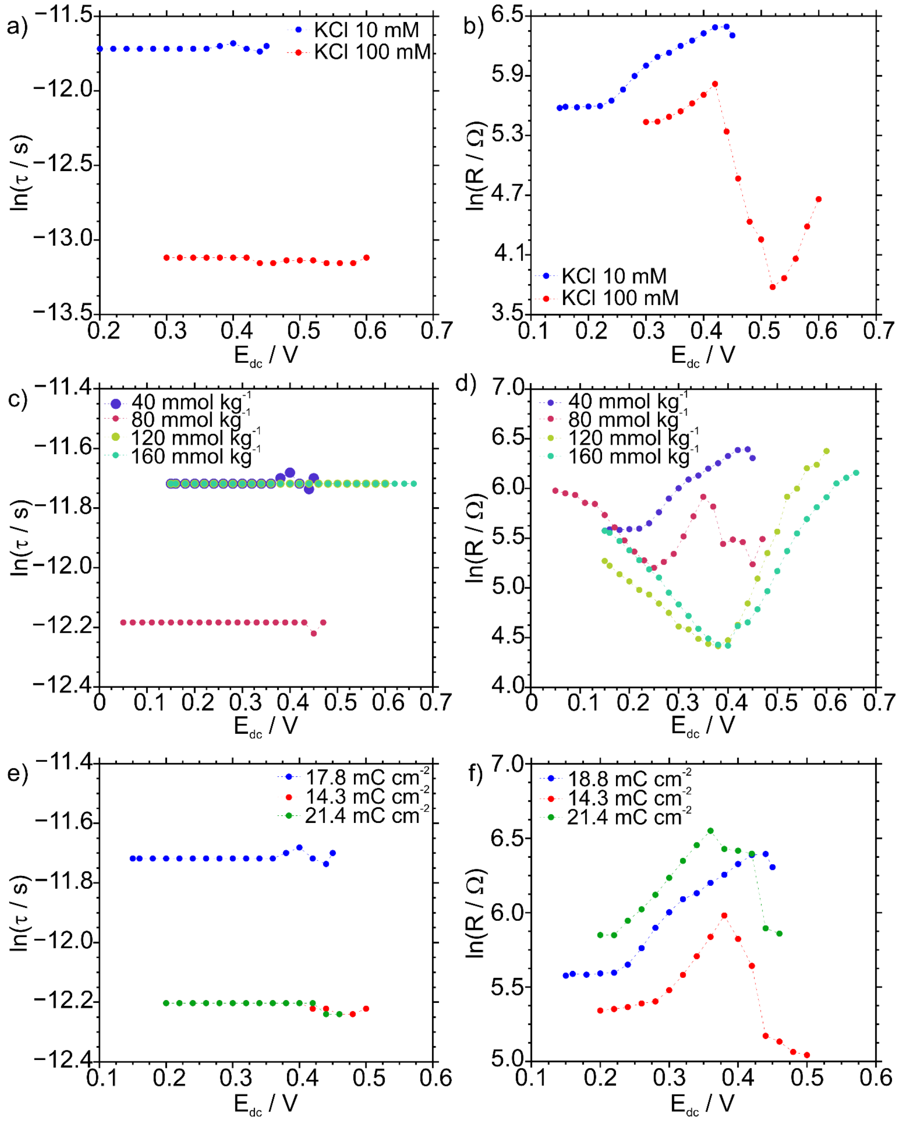

Figure S9. For Peak I, plot of the logarithm of the relaxation time values (left) and resistance (right) as a function of the E_{dc} while changing: **(a,b)** the electrolyte concentration, **(c,d)** the $TFPB^-$ concentration in the membrane and **(e,f)** the applied charge density in the electropolymerization of the POT layer.

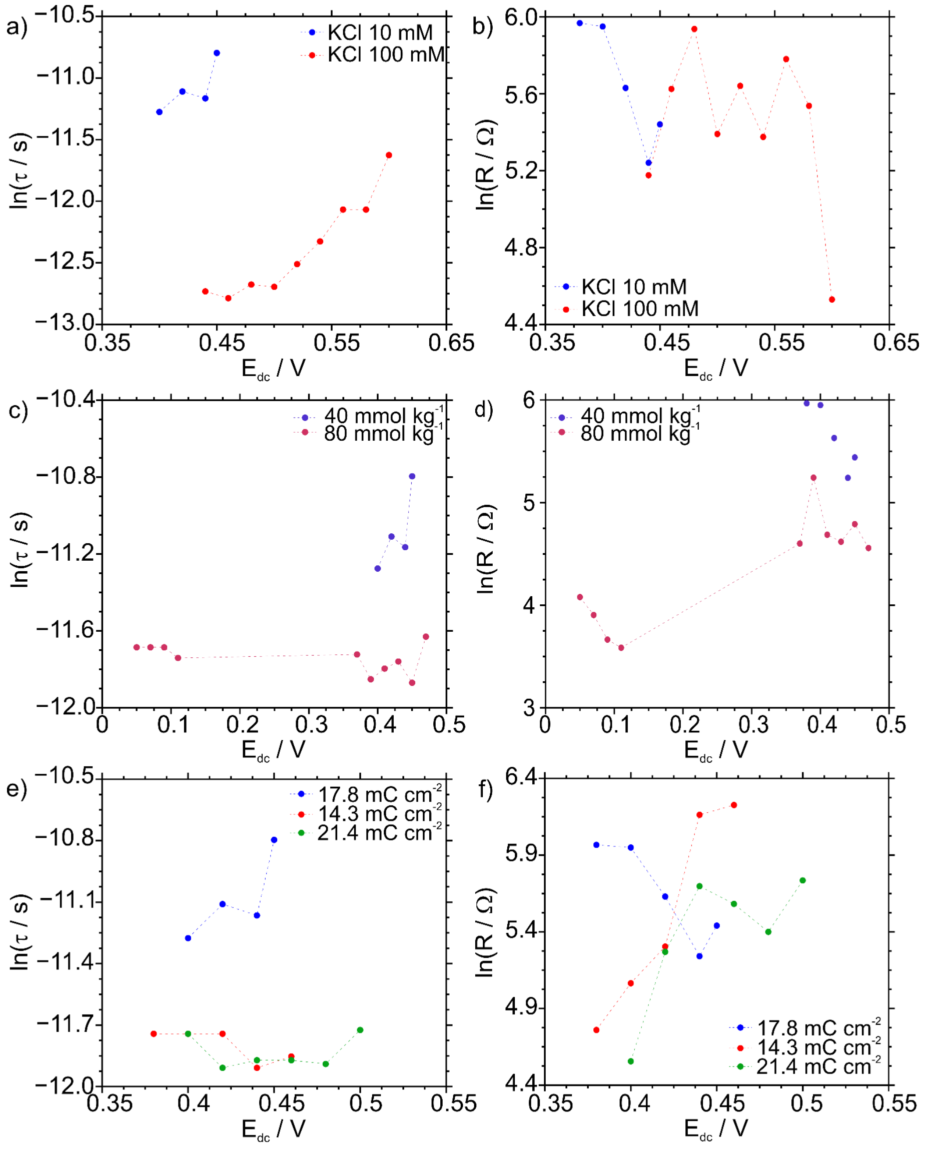

Figure S10. For Peak II, plot of the logarithm of the relaxation time values (left) and resistance (right) as a function of the E_{dc} while changing: **(a,b)** the electrolyte concentration, **(c,d)** the TFPB^{-} concentration in the membrane and **(e,f)** the applied charge density in the electropolymerization of the POT layer.

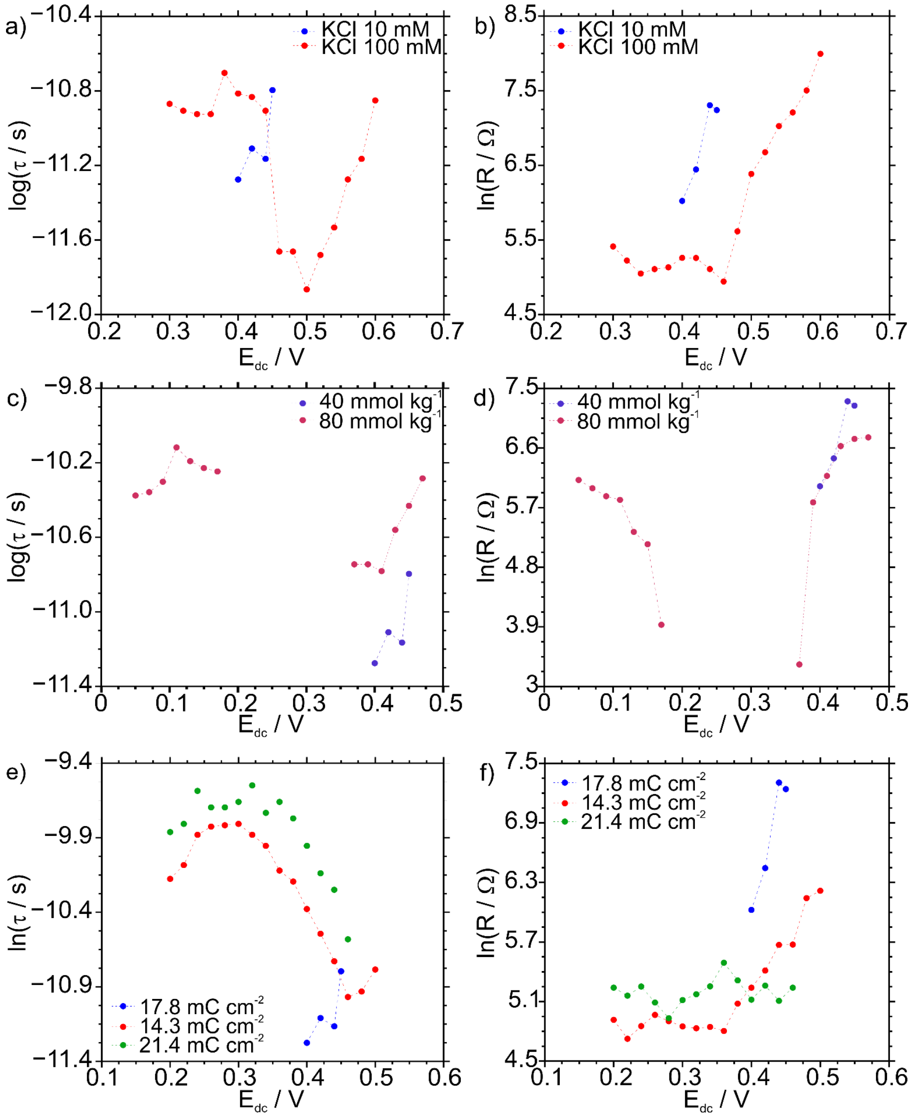

Figure S11. For Peak III, plot of the logarithm of the relaxation time values (left) and resistance (right) as a function of the E_{dc} while changing: **(a,b)** the electrolyte concentration, **(c,d)** the TFPB⁻ concentration in the membrane and **(e,f)** the applied charge density in the electropolymerization of the POT layer.

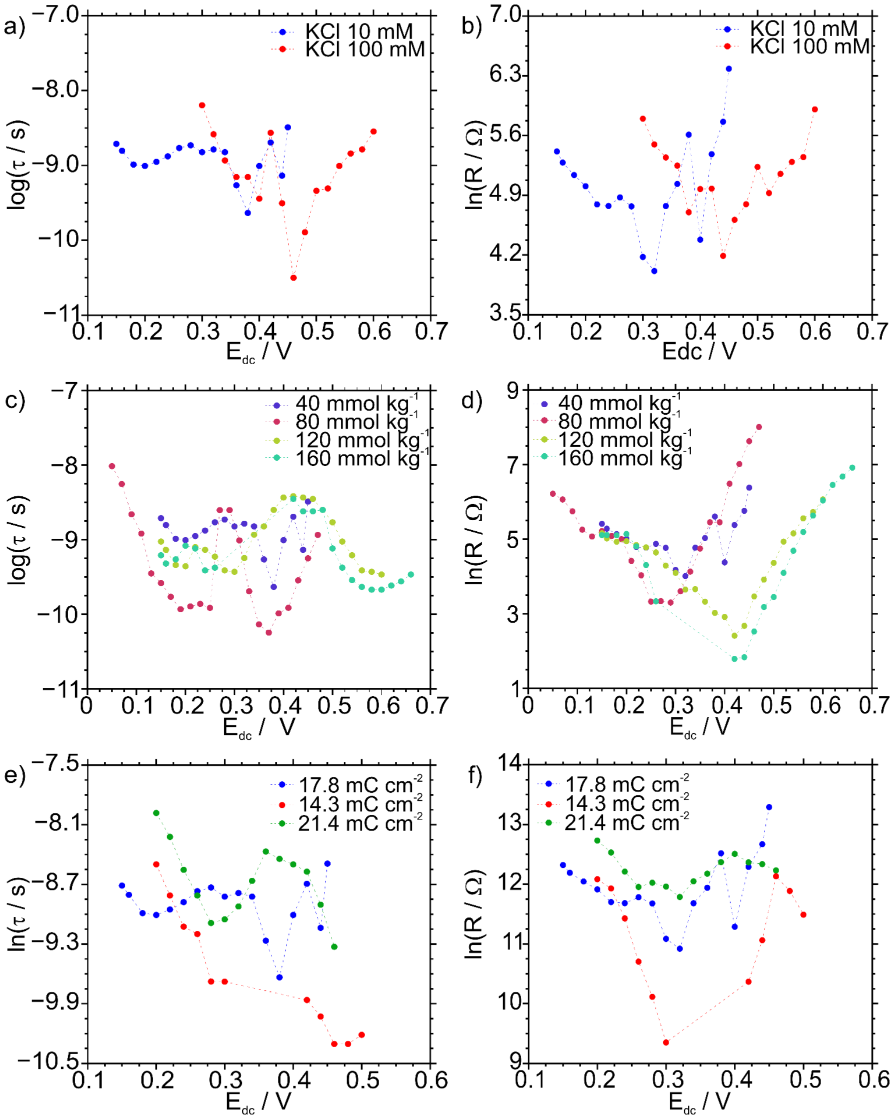

Figure S12. For Peak IV, plot of the logarithm of the relaxation time values (left) and resistance (right) as a function of the E_{dc} while changing: **(a,b)** the electrolyte concentration, **(c,d)** the $TFPB^-$ concentration in the membrane and **(e,f)** the applied charge density in the electropolymerization of the POT layer.

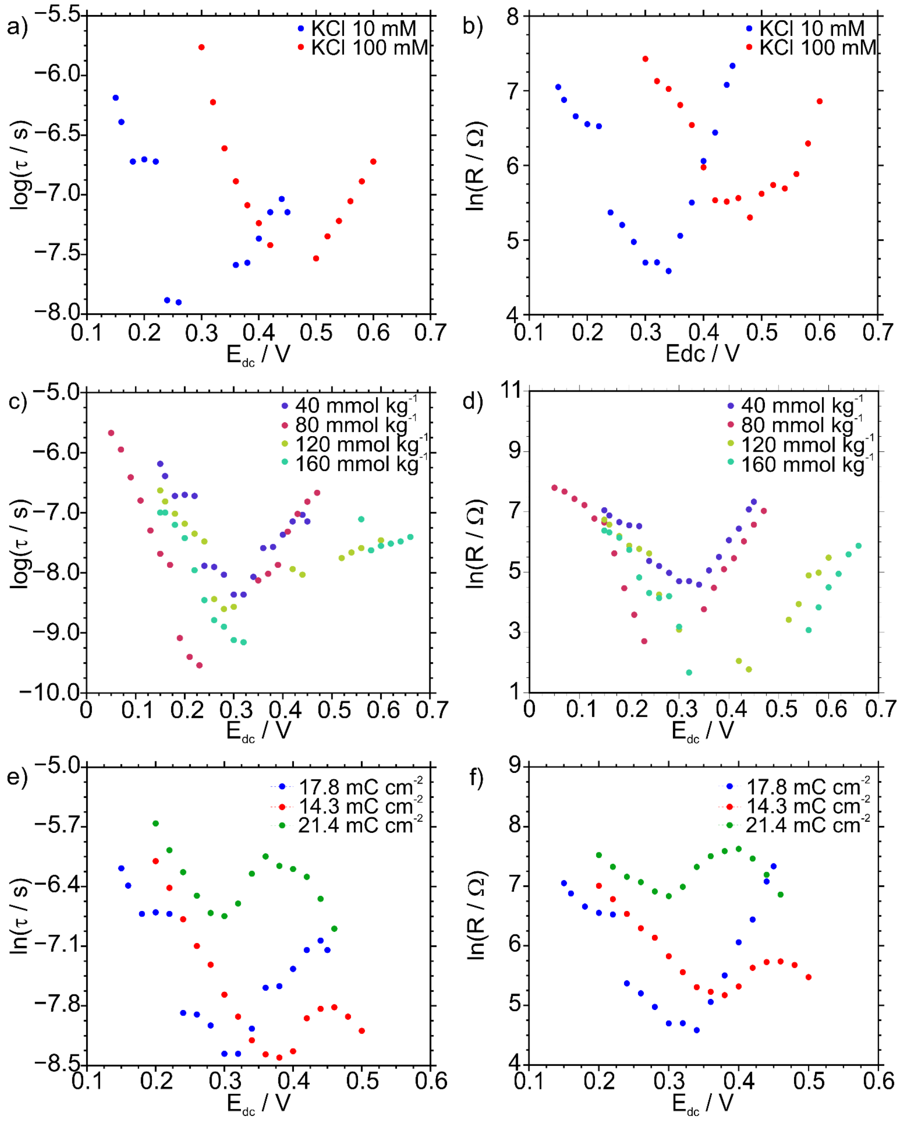

Figure S13. For Peak V, plot of the logarithm of the relaxation time values (left) and resistance (right) as a function of the E_{dc} while changing: **(a,b)** the electrolyte concentration, **(c,d)** the TFPB^{-} concentration in the membrane and **(e,f)** the applied charge density in the electropolymerization of the POT layer.

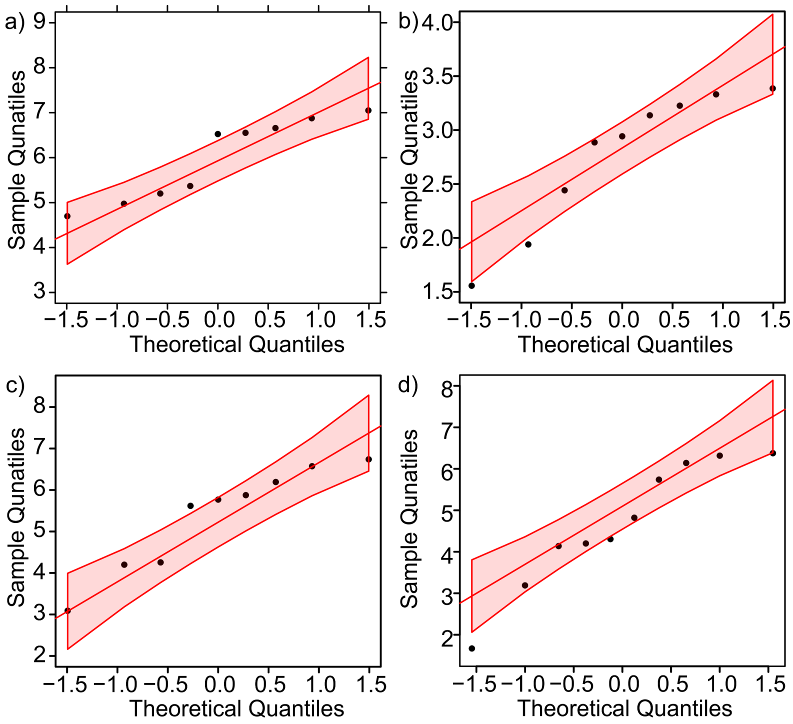

Figure S14. Quantile-Quantile plot for Peak V at varying TFPB⁻ concentration in the membrane: a) 40, b) 80, c) 120 and d) 160 mmol kg^{-1}. Sample quantiles shows the empirically observed quantiles that correspond to the logarithm of the resistance for peak V as a function of the theoretical quantiles that correspond to the quantiles expected from a normal distribution with the same mean and variance as the empirical distribution. The shadow area represents a confidence interval of 95%.

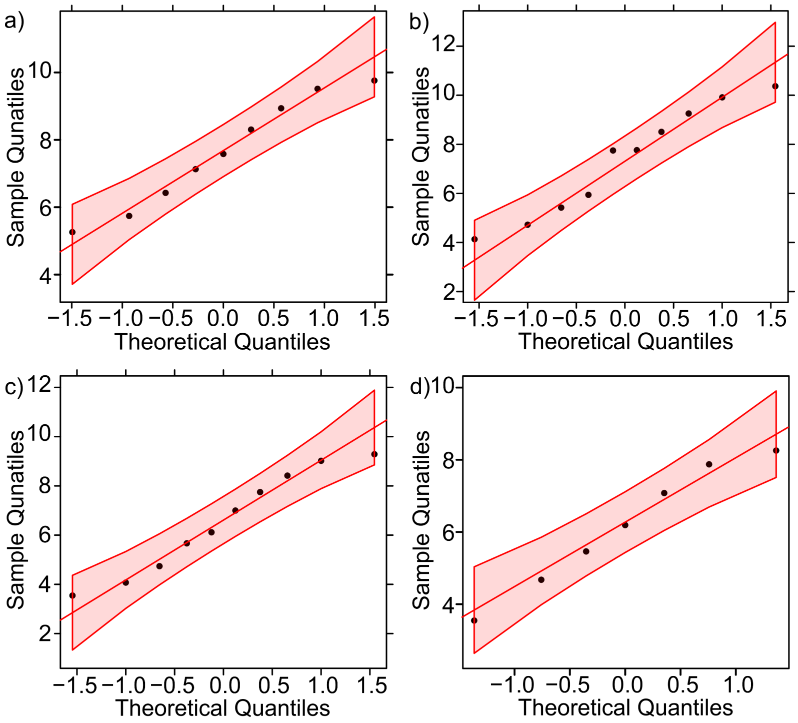

Figure S15. Quantile-Quantile plot for Peak VI at varying TFPB^{-} concentrations in the membrane: a) 40, b) 80, c) 120 and d) 160 mmol kg^{-1}. Sample quantiles shows the empirically observed quantiles that correspond to the logarithm of the resistance for Peak VI as a function of the theoretical quantiles that correspond to the quantiles expected from a normal distribution with the same mean and variance as the empirical distribution. The shadow area represents a confidence interval of 95%.

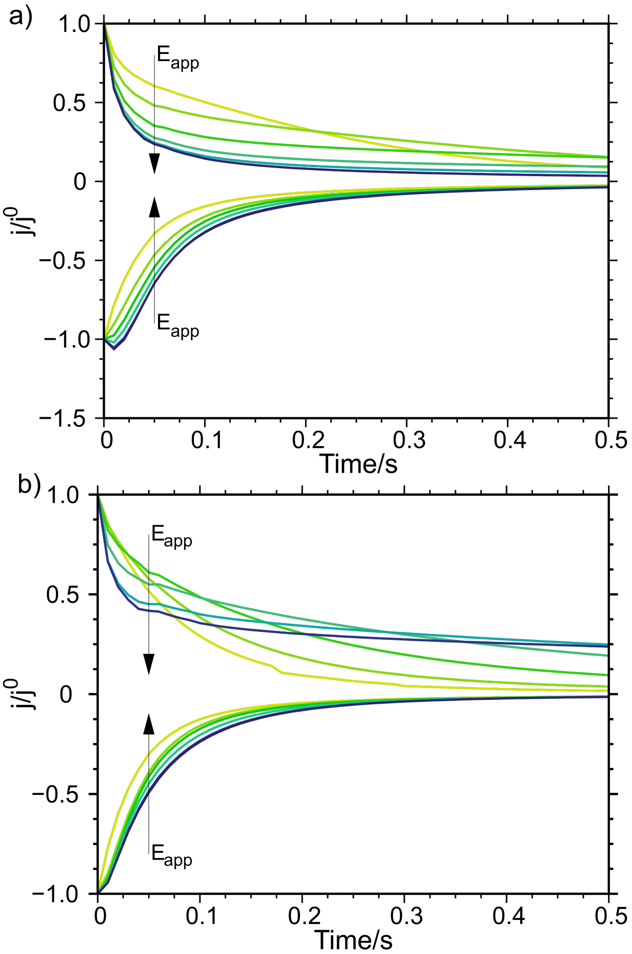

Figure S16. Chronoamperometric responses of a POT film coated with a membrane composed of (a) 40 mmol kg^{-1} of $TFPB^{-}$ and (b) 120 mmol kg^{-1} of $TFPB^{-}$ at different applied potentials approaching to the voltammetric E_{peak}. For the oxidation process: 380 mV, 340 mV, 300 mV, 260 mV, 220 mV and 150 mV for 40 mmol kg^{-1} and 380 mV, 340 mV, 300 mV, 260 mV, 220 mV and 200 mV for 120 mol kg^{-1}. The aqueous solution was 10 mM KCl.

References

(1) Otero, T. F.; Santos, F. Polythiophene Oxidation: Rate Coefficients, Activation Energy and Conformational Energies. *Electrochimica Acta* **2008**, *53* (7), 3166–3174.

(2) Sandoval, A. P.; Feliu, J. M.; Torresi, R. M.; Suárez-Herrera, M. F. Electrochemical Properties of Poly(3,4-Ethylenedioxythiophene) Grown on Pt(111) in Imidazolium Ionic Liquids. *RSC Adv* **2014**, *4* (7), 3383–3391.
